# Mechanistic safety assessment via multi-omic characterisation of systemic pathway perturbations following in vivo MAT2A inhibition

**DOI:** 10.1007/s00204-024-03771-w

**Published:** 2024-05-17

**Authors:** Valentina Fogal, Filippos Michopoulos, Andrew F. Jarnuczak, Ghaith M. Hamza, Stephanie Harlfinger, Paul Davey, Heather Hulme, Stephen J. Atkinson, Piotr Gabrowski, Tony Cheung, Michael Grondine, Clare Hoover, Jonathan Rose, Chandler Bray, Alison J. Foster, Sean Askin, Muntasir Mamun Majumder, Paul Fitzpatrick, Eric Miele, Ruth Macdonald, Hector C. Keun, Muireann Coen

**Affiliations:** 1 Oncology Safety, Safety Sciences, Clinical Pharmacology & Safety Sciences, R&D, AstraZeneca, Cambridge, UK; 2 Bioscience, Research and Early Development, Oncology R&D, AstraZeneca, Cambridge, UK; 3 Data Sciences & Quantitative Biology, Discovery Sciences, R&D, AstraZeneca, Cambridge, UK; 4 Discovery Biology, Discovery Sciences, R&D, AstraZeneca, R&D Boston, Waltham, USA; 5 DMPK, Oncology R&D, AstraZeneca, Cambridge, UK; 6 Chemistry, Oncology R&D AstraZeneca, Cambridge, UK; 7 Imaging and Data Analytics, Clinical Pharmacology and Safety Sciences, R&D, AstraZeneca, Cambridge, UK; 8 Biological Insights Knowledge Graph, R&D IT, AstraZeneca, Barcelona, Spain; 9 Oncology R&D, AstraZeneca, R&D Boston, Waltham, USA; 10 Oncology Safety Pathology, Clinical Pharmacology & Safety Sciences, R&D, AstraZeneca, R&D Boston, Waltham, USA; 11 Animal Science & Technologies, R&D, AstraZeneca, Cambridge, UK; 12 Regulatory Toxicology and Safety Pharmacology, Clinical Pharmacology & Safety Sciences, R&D, AstraZeneca, Cambridge, UK; 13 Advanced Drug Delivery, Pharmaceutical Sci, R&D, AstraZeneca, Cambridge, UK; 14 Safety Sciences, Clinical Pharmacology & Safety Sciences, R&D, AstraZeneca, Gothenburg, Sweden; 15 Cancer Metabolism & Systems Toxicology Group, Division of Cancer, Department of Surgery and Cancer, Imperial College London, London, UK

**Keywords:** One-carbon metabolism, MAT2A enzyme, Drug safety, Methionine metabolism, Metabolomics, Multi-omics

## Abstract

The tumour suppressor p16/CDKN2A and the metabolic gene, methyl-thio-adenosine phosphorylase (MTAP), are frequently co-deleted in some of the most aggressive and currently untreatable cancers. Cells with MTAP deletion are vulnerable to inhibition of the metabolic enzyme, methionine-adenosyl transferase 2A (MAT2A), and the protein arginine methyl transferase (PRMT5). This synthetic lethality has paved the way for the rapid development of drugs targeting the MAT2A/PRMT5 axis. MAT2A and its liver- and pancreas-specific isoform, MAT1A, generate the universal methyl donor S-adenosylmethionine (SAM) from ATP and methionine. Given the pleiotropic role SAM plays in methylation of diverse substrates, characterising the extent of SAM depletion and downstream perturbations following MAT2A/MAT1A inhibition (MATi) is critical for safety assessment. We have assessed in vivo target engagement and the resultant systemic phenotype using multi-omic tools to characterise response to a MAT2A inhibitor (AZ’9567). We observed significant SAM depletion and extensive methionine accumulation in the plasma, liver, brain and heart of treated rats, providing the first assessment of both global SAM depletion and evidence of hepatic MAT1A target engagement. An integrative analysis of multi-omic data from liver tissue identified broad perturbations in pathways covering one-carbon metabolism, trans-sulfuration and lipid metabolism. We infer that these pathway-wide perturbations represent adaptive responses to SAM depletion and confer a risk of oxidative stress, hepatic steatosis and an associated disturbance in plasma and cellular lipid homeostasis. The alterations also explain the dramatic increase in plasma and tissue methionine, which could be used as a safety and PD biomarker going forward to the clinic.

**Supplementary Information:**

The online version contains supplementary material available at 10.1007/s00204-024-03771-w.

## Introduction

MTAP encodes the metabolic enzyme methyl-thio-adenosine phosphorylase; it is homozygously deleted in 15% of cancers, and frequently co-deleted with the tumour suppressor CDKN2A at the 9p.21 genomic locus (Zhang et al. 1996). This genetic variant is a well-known negative prognostic factor in cancer (Zhao et al. 2016), yet there are no therapies that selectively target CDKN2A/MTAP-deleted tumours. Genetic screens in MTAP-deleted cells have identified two genes, PRMT5 and MAT2A that are synthetically lethal with MTAP loss (Kryukov et al. 2016; Marjon et al. 2016; Mavrakis et al. 2016). MTAP deletion leads to the accumulation of its substrate, MTA (methylthioadenosine), which partially inhibits the activity of PRMT5, while other methyltransferases are relatively unaffected (Marjon et al. 2016). Inhibition of PRMT5 activity results in a reduction in SDMAs (symmetrically di-methylated arginine residues) on target proteins, many of which are involved in mRNA splicing, gene expression or DNA repair (Kalev et al. 2021). This creates a selective dependency on MAT2A, as this enzyme is responsible for the synthesis of the universal methyl group donor S-adenosylmethionine (SAM), the key metabolite required for the methylation function of PRMT5. Novel therapeutic approaches against MTAP-deleted tumours aim to target PRMT5 either directly or indirectly via inhibition of MAT2A enzymatic function. At present, several clinical candidates exploit the MAT2A/PRMT5 target space, with small molecule inhibitors of both MAT2A and PRMT5 currently being assessed in clinical trials (Agios Pharmaceuticals, AACR-NCI-EORTC 2019)(Atkinson et al. 2022; Guo et al. 2022; Marjon et al. 2021).

MTAP and MAT2A reside in an interconnected network of metabolic pathways (methionine cycle, folate cycle and trans-sulfuration pathway) known as one-carbon (1-C) metabolism, which generates metabolites for critical cellular processes (Locasale 2013). Among them, the metabolism of the essential amino acid methionine is pivotal; consequently, it influences many biological processes in both normal and cancer cells (Sanderson et al. 2019). MTAP participates in the methionine salvage pathway through cleavage of the by-product of polyamine synthesis, MTA, to adenine and MTR-1-P (methyl-thioribose-1-phosphate), which leads to methionine regeneration. MAT2A, on the other hand, produces the universal methyl donor, SAM, from methionine. Intracellular transmethylation reactions utilize SAM to modulate gene expression via methylation of DNA, RNA and histones (Reid et al. 2017). SAM is also used for methylation of non-histone proteins (Levy 2019) and lipids (Vance 2014), and therefore, regulates critical cellular processes and signalling pathways at the post-translational level. In addition, SAM is required for the synthesis of polyamines which contribute to the stability of RNA, DNA and proteins. Furthermore, the by-product of methylation reactions, SAH (S-adenosylhomocysteine), is converted into homocysteine, which is then transformed back to methionine by 5-methyltetrahydrofolate-homocysteine methyl transferase through a methyl group donation from the folate cycle. Homocysteine also serves as a precursor for the synthesis of cysteine, glutathione and taurine through the trans-sulfuration pathway. As such, alterations in methionine metabolism and methionine derivatives (SAM in particular) can affect a plethora of cell functions (Sanderson et al. 2019).

The impact of MAT2A inhibition on normal tissues is currently unclear, as is understanding of the extent of systemic SAM depletion following MAT2A inhibition. There are three MAT genes; MAT1A and MAT2A encode catalytic isoforms that share a high degree of amino acid sequence identity but have differential tissue expression; MAT1A is specifically expressed in the liver and pancreas whereas MAT2A is expressed in extra-hepatic tissues as well as non-parenchymal cells of the liver (eg hepatic stellate and Kupffer cells) (Lu et al. 2003; Murray et al. 2019). Multimers of these enzymes differ in substrate kinetic properties (Maldonado et al. 2018). A third gene, MAT2B, unrelated in amino acid sequence to MAT1A/2A, encodes a MAT2A regulatory protein (Nordgren et al. 2011).

Liver homeostasis may be particularly sensitive to MAT2A inhibition for several reasons; methionine metabolism occurs predominantly in the liver where up to 85% of all methylation reactions take place (Finkelstein 1990). MAT1A knockout mice spontaneously develop macrovesicular steatosis and increased liver proliferation, which progresses to hepatocellular carcinoma (Alonso et al. 2017; Cano et al. 2011; Lu et al. 2001). In addition, MAT1A KO mice are more likely to develop fatty liver disease when fed a choline-depleted diet (Lu et al. 2001). Furthermore, the selectivity of existing MAT2A inhibitors for MAT1A has not been defined, and this is of key importance for drug safety assessment, as inhibition of both MAT1A and MAT2A in the liver would likely elicit greater steatosis than knockout of MAT1A alone since MAT2A is known to compensate for MAT1A loss (Lu et al. 2001). Depletion of dietary methionine and choline has long been utilised as a model of hepatic steatosis, as it impairs de novo synthesis of phosphatidylcholine, the major phospholipid essential for the formation of very-low-density lipoprotein (VLDL) (Oz et al. 2008; Yao and Vance 1988, 1990). Similarly, MAT1A deletion impairs VLDL assembly and secretion as well as plasma lipid homeostasis in mice (Cano et al. 2011).

The MAT2A inhibitor, AG-270, was shown in a Phase 1 clinical trial to cause hyperbilirubinemia (proposed to be as a result of UGT1A1 inhibition) and reversible, acute liver injury as a dose limiting toxicity although the mechanism has not been elucidated (Agios Pharmaceuticals, AACR-NCI-EORTC 2019). Given the above, and the requirement for methionine and SAM in several fundamental biologic processes, we performed a systems toxicology analysis to better understand the safety implications of MAT2A inhibition.

Using a well-characterised tool MAT2A inhibitor (AZ’9567 or MAT2Ai) (Atkinson et al. 2024), we performed an in vivo rat study and conducted multi-omic (metabolomic, transcriptomic, proteomic and lipidomic) assessment of liver, which was anchored with histopathology and clinical chemistry analysis. Our study characterised the consequences of MATi at a systems level and highlighted a number of safety concerns together with monitorable, circulating safety biomarkers.

## Materials and methods

### Animals and dosing

All in vivo experimental procedures were conducted in accordance with United Kingdom legislation (Animals (Scientific Procedures) Act, 1986) and were compliant with the ARRIVE guidelines. 8–10 week old male (210–230 g) Crl:WI Han Wistar rats (Charles River Laboratories Ltd, UK) were acclimatised to the facility for 10 days and then randomised to groups based on bodyweight. The studies involved vehicle control (*n* = 5) and AZ’9567 inhibitor treatment (*n* = 5 per dose level).

Animals were treated via oral gavage with vehicle control (5% (v/v) DMSO/45% v/v (60% w/v SBE-β-CD in purified water)/50% v/v (20% w/v PVP K30 in purified water) and AZ’9567 (3, 10, 30 mg/kg BID). All animals were dosed twice daily (BID) with a dose volume of 10 mL/kg for 7 days, with the final dose being administered on the morning of Day 8. The dose interval between the first and second dose on each day was 8 h. These doses were selected as they showed robust anti-tumour and PD modulation (SDMA and SAM) in a murine MTAP KO HCT116 xenograft model (Atkinson et al. 2024). Animals were terminally anaesthetised via administration of isofluorane, and euthanised via exsanguination at 4 h post the final dose on day 8.

### Plasma toxicokinetic analysis

A whole blood sample (32 µL) was collected via a tail vein prick into a K2EDTA treated capillary tube, centrifuged at 1,500 g for 10 min at 4 °C, and plasma collected using a micropipette (non-EDTA Ref.no. 172292, Vitrex Medical A/S, Denmark). Samples were collected on Day 1 and Day 7 at 1 h, 2 h, 4 h, 8 h (pre-second dose), 10 h and 24 h post the first dose. AZ’9567 plasma concentrations were quantified using High Performance Liquid Chromatography coupled with tandem Mass Spectrometry as described in supplementary M&M. Free plasma concentrations of AZ’9576 were derived by multiplying total concentrations with rat plasma fraction unbound of 0.00614, which was derived in a separate in vitro experiment via rapid equilibrium dialysis.

### Liver histopathology

Following euthanasia and blood sample collections, animals were necropsied and subjected to gross examination. Samples of liver, brain and heart were collected and fixed in 10% neutral buffered formalin at room temperature for 48 h, followed by routine histology processing (Leica Biosystems) to paraffin embedded wax blocks. Paraffin embedded tissues were cut into 5 µM sections using a microtome (Leica Biosystems) and mounted on glass microscope slides. Tissue sections were stained with hematoxylin and eosin and coverslipped. Stained slides were digitally scanned using an AT2 Scanscope pathology slide scanner (Leica Biosystems) and whole slide images were reviewed by a veterinary pathologist.

### Clinical chemistry

A whole blood sample (2.0 mL) was collected into lithium heparin from the caudal vena cava under isoflurane/oxygen anaesthesia immediately prior to euthanasia. Blood samples were centrifuged for 10 min at 4 °C to separate the plasma fraction. Plasma samples were divided into 0.5 mL aliquots and frozen at 20 °C for storage prior to chemistry analysis. Plasma chemistry analysis was performed by Charles River Laboratories (Edinburgh, Scotland) following standard procedures.

### Determination of SAM and methionine from plasma and various tissues

SAM and methionine levels were quantified in plasma samples and tissues using Ultra Performance Liquid Chromatography (UPLC) coupled with quantitative MS in ESI positive ion mode as described in Supplementary M&M.

### Metabolite extraction and mass spectrometry analysis

Tissue material was extracted into two different metabolome fractions, one for polar metabolites, generated after extraction with ACN/MeOH/ H_2_O 40/40/20 v/v/v, and a lipidome fraction obtained with Butanol/MeOH 3/1 v/v. Further details of the wider metabolome extraction and analysis parameters can be found in supplementary M&M.

Polar metabolite raw spectrometric data was processed with MultiQuant 3.0.3 (ABSCIEX) or Tracefinder 5.1 (Thermo Scientific) for negative and positive mode data sets, respectively, using in house-built retention time libraries of pure authentic standards. Metabolite peak areas were then imported to an Excel spreadsheet for log(2) transformation and median fold change normalisation. Lipidomic data were mined and deconvoluted using free access software MSDIAL 5.1.2 (RIKEN, Japan). For putative lipid identification, SpectralAtlas VS69 was used on positive and negative mode data sets.

### Proteome sample preparation

Liver tissue were homogenized in PreOmics iST buffer (PreOmics) using a bead ruptor. Protein was subsequently reduced, alkylated and proteolytically digested into peptides. Peptides were purified using PreOmics iST cartridges and vacuum-centrifuged to dryness. Peptides were subsequently reconstituted in 2 vol% ACN and 0.1 vol% formic acid for single-run LC–MS analysis. Peptides were analyzed using a Thermo EASY-nLC1200 coupled online to an Exploris 480 Mass Spectrometer equipped with a Nanospray Flex Ion Source integrated with a column oven (PRSO-V1, Sonation) and a FAIMS unit. Peptides were separated using a nonlinear gradient and analyzed by acquiring spectra through Data Independent Acquisition (DIA). Data was analyzed using Spectronaut V15.7.2 (Biognosys AG) direct DIA analysis using an isoform Rat database (UniProt downloaded 22.02.2021) consisting of 9,746 protein entries utilizing the Pulsar search engine. Analysis settings were maintained to factory settings and identification was set to 1% false discovery rate (FDR) for precursor and protein level. Additional information is provided in Supplementary M&M**.**

### Liver transcriptomics

Total RNA was purified from liver samples with RNAdvance Tissue^™^ kit by Beckman Coulter Life Sciences according to manufacturer’s instructions. The method was carried out in an automated robotic solution with Biomek i7 Hybrid^™^. The concentration and quality of isolated RNA molecules were measured applying parallel capillary electrophoresis using Agilent Fragment Analyzer 5300 (DNF-471-33-SS Total RNA 15nt). The data were analysed in ProSize data analysis software to derive the concentration and RNA quality numbers (RQN) for individual samples.

The double-stranded cDNA obtained with Poly (A)-mRNA enrichment (500 ng total RNA input) was subjected to library preparation using KAPA Hyper Prep Kit (Kapa Biosystems, MA, USA) according to manufacturer’s protocol. The libraries were pooled in a single library and were sequenced on an Illumina NovaSeq6000 with a v1.5 SP 100 cycle kit as paired-end reads to PE51bp. The sequencing runs were carried out according to the manufacturer’s instructions.

### Data analysis

Data analysis was performed using R Statistical Software (version 4.3.0). Proteomics, transcriptomics and metabolomics expression changes were visualised using heatmaps showing log_2_ fold change relative to control. In the heatmaps missing values were highlighted in grey.

### Secondary pharmacology

Secondary pharmacology assays were performed at Eurofins using standard experimental techniques against a total of 86 targets across a broad pharmacological space. Assay details along with experimental protocols are described at https://www.eurofinsdiscoveryservices.com. Radioligand binding assays were used to assess the ability of AZ’9567 to interact with G-protein coupled receptors (GPCRs), ion channels and transmembrane transporters. Assays measuring substrate turnover or phosphorylation by isolated proteins were used for kinase and enzyme targets, allowing direct determination of the mode of action of the compounds. Where binding activity was observed at a GPCR target, mode of action was determined using cell based functional assays with secondary messenger read outs. Assays were run in eight points concentration response mode with half log dilutions and IC_50_, EC_50_ or K_i_ (Cheng and Prusoff 1973) values determined.

## Results

### In vitro and in vivo characterisation of MAT2A inhibitor AZ’9567

To understand the in vivo implications of MAT2A inhibition, we used a tool MAT2A inhibitor AZ’9567. The structure and in vitro properties of the compound are shown in Table 1 together with potency in the HCT116 isogenic cell line (MTAP WT and KO) and in the MAT2A biochemical assay (SAM endpoint).

**Table 1:** Structure and in vitro properties of AZ’9567

Structure	MW	LogD (pH 7.4)	Biochemical MAT2A Rapidfire pIC_50_	HCT116 cell SDMA pIC_50_ MTAP KO/Parental	HCT116 cell SAM MTAP KO pIC_50_
	447.4	3.4	9.1	8.6/5.5^a^	8.9

^a^*n* = 11, partial responses < 75% S_inf_ excluded from mean calculation, also tested < 5.5, *n* = 18

As previously described (Atkinson et al. 2024), secondary pharmacology profiling revealed good off target selectivity when tested against a total of 86 targets. Secondary pharmacology screening for AZ’9567 (SI Table 1) showed measurable activity (K_i_, IC_50_) against 12 off-targets below 10 µM with < 1 µM potency observed at one of the targets (adenosine transporter, antagonism). Exposure in the rat in vivo study following BID dosing of AZ’9567 for 7 days did not provide ‘cover’ over these off-targets (Secondary pharmacology profile SI Fig. 1). We also characterised our MAT2Ai in a broad suite of in vitro cell based assays representing hepatic, cardiac, mitochondrial and general cytotoxicity, which revealed a lack of effect in all assays (SI Table 2).

AZ’9567 was, therefore, used to assess the safety of MAT2A inhibition in a 7-day-rat investigative safety study with treatment at 3 dose levels (3, 10, 30 mg/kg BID) of AZ’9567 (or vehicle control).

**Fig. 1:**
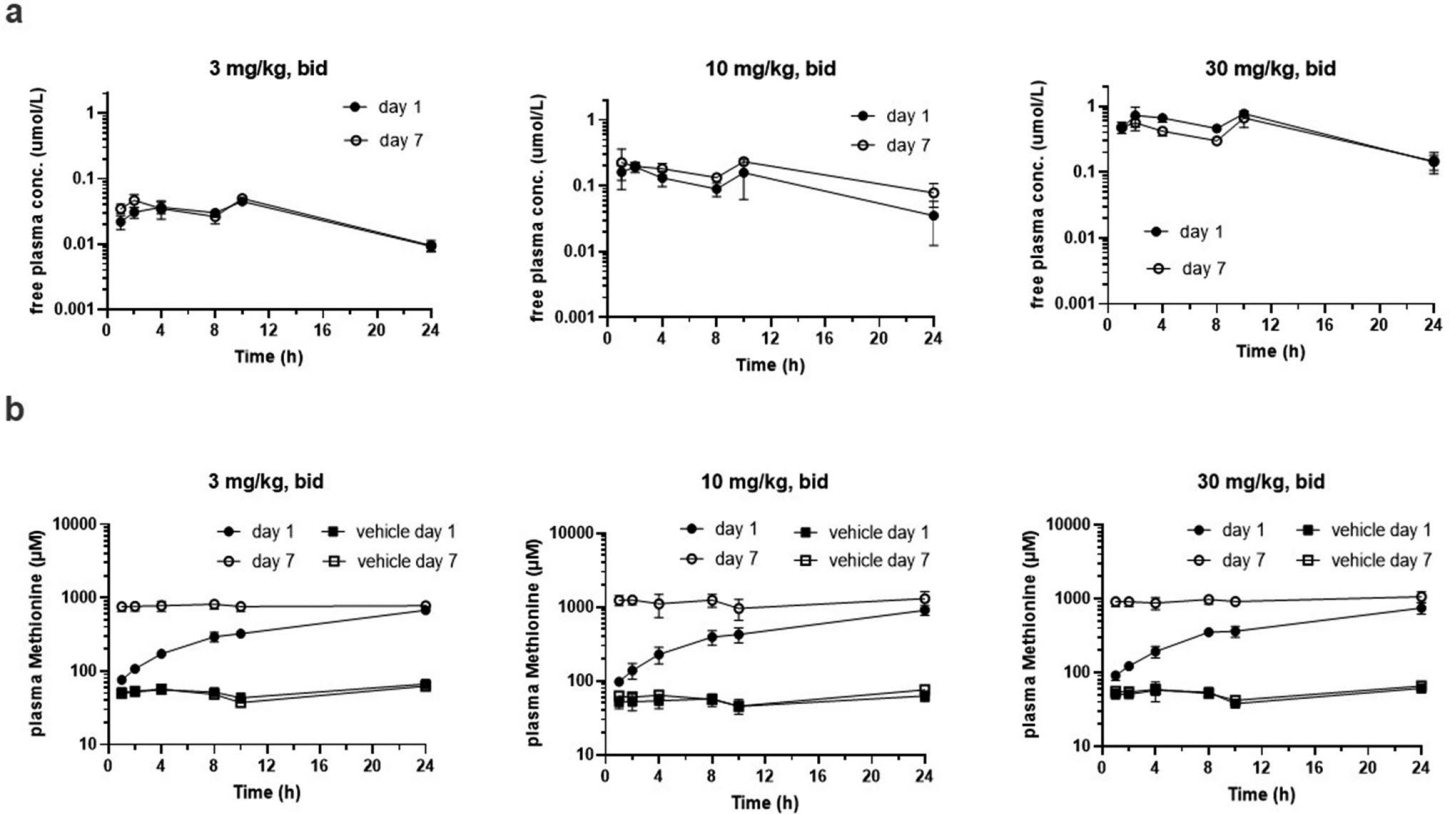
In vivo plasma PKPD. Plasma concentration vs time profiles for AZ’9567 (**a**) and methionine (**b**) on day 1 and day 7 following oral twice daily dosing of AZ’9567 to male Han Wistar rats. Mean, SD, *n* = 5

The free plasma concentration vs time profiles of AZ’9567 obtained on day 1 and day 7 of dosing are shown in Fig. 1a. Free AUC_24h_ and C_max_ observed on day 1 and day 7 were constant and increased roughly in proportion to the dose (Table 2). Mean free plasma concentrations observed at 24 h (C_min_) following 3, 10, 30 mg/kg BID oral dosing covered the MAT2A biochemical IC_50_ 9-, 35-, 143-fold, respectively, on day 1 and 9-, 78-, 147- fold, respectively, on day 7.

**Table 2:** Pharmacokinetic results following twice daily oral dosing of AZ’9567 to rats

AZ’9567	3 mg/kg, BID		10 mg/kg, BID		30 mg/kg, BID	
	day 1	day 7	day 1	day 7	day 1	day 7
Free AUC_24h_ (µM*h)	0.69 ± 0.07	0.76 ± 0.11	2.6 ± 1.1	3.9 ± 0.70	12 ± 1.1	9.9 ± 2.2
Free C_max_ (µM)	0.04 ± 0.004	0.05 ± 0.01	0.21 ± 0.06	0.27 ± 0.11	0.87 ± 0.19	0.68 ± 0.19
Free C_min24h_ (µM)	0.01 ± 0.001	0.01 ± 0.002	0.04 ± 0.02	0.08 ± 0.03	0.14 ± 0.04	0.15 ± 0.05
Fold cover of ^1^MAT2A biochemical IC_50_ at mean free C_min24h_	9	9	35	78	143	147

Mean, SD, *n* = 5

^1^as shown in Table 1

Methionine plasma concentrations were simultaneously quantified in our micro-capillary TK sample, covering a 24 h kinetic profile on day 1 and day 7 of vehicle treatment and were found to be at a constant baseline (Fig. 1b) of 53 ± 10 µmol/L (*n* = 90 data-points) on day 1 and 55 ± 11 µmol/L (n = 88) on day 7 across all three control groups. Methionine plasma concentrations increased over the course of 24 h following BID treatment with AZ’9567 on day 1 reaching similar 24 h levels across all three dose groups of 777 ± 110 µmol/L (3 mg/kg, BID), 1296 ± 321 µmol/L (10 mg/kg, BID) and 1061 ± 170 µmol/L (30 mg/kg, BID) (Fig. 1b). On day 7 of treatment, methionine plasma concentrations were again found to be at a constant baseline over the 24 h kinetic achieving concentrations of 775 ± 104 µmol/L (3 mg/kg, BID), 1180 ± 279 µmol/L (10 mg/kg, BID) and 940 ± 142 µmol/L (30 mg/kg, BID) (Fig. 1b and Table 3). Oral twice daily dosing of AZ’9567 increased the average methionine plasma concentration by 15-, 19- and 17-fold for the 3, 10 and 30 mg/kg dose groups, respectively.

**Table 3:** Average methionine plasma concentrations (µmol/L) at baseline and after twice daily oral administration of AZ’9567 for 7 days

Dose groups	Day 1	Day 7
Vehicle 3 mg/kg, po, bid	54 ± 8.4 777±110	51 ± 8.9 775 ± 104
Vehicle 10 mg/kg, po, bid	54 ± 10 1296 ± 321	61 ± 11 1180 ± 279
Vehicle 30 mg/kg, po, bid	52 ± 11 1061 ± 170	55 ± 10 940 ± 142

Mean, SD, *n* = 30, *n.c.* not calculated

Efficacy data were generated in HCT116 KO xenograft murine models (Atkinson et al. 2024) with robust anti-tumour response demonstrated at 20 mg/kg BID with > 90% depletion of both SAM and SDMA on day 24.

We had no clinical observations during the in-life phase of the study. A significant drop in both food consumption and body weight was recorded with all dose groups, however, these changes had a minimal impact on the liver metabolome and proteome when compared to the treatment (SI Fig. 2).

Microscopic examination of the liver from animals dosed with 30 mg/kg MAT2Ai revealed only a minimal decrease in hepatocyte glycogen stores when compared with vehicles (SI Fig. 3a), consistent with decreased food intake. No microscopic abnormalities were observed on histopathological examination of the heart or brain, however, dose-dependent pancreatic necrosis resulting in loss of both exocrine and endocrine parenchyma, and bone marrow toxicity affecting mostly the erythroid compartment were observed (data not shown). We will not discuss these lesions any further in this manuscript as further studies are on-going to delineate the mechanism of toxicity and if on- or off-target mediated. Plasma chemistry analysis revealed no differences in markers of liver injury or cholesterol levels between vehicle and MAT2Ai treated animals.

**Fig. 2:**
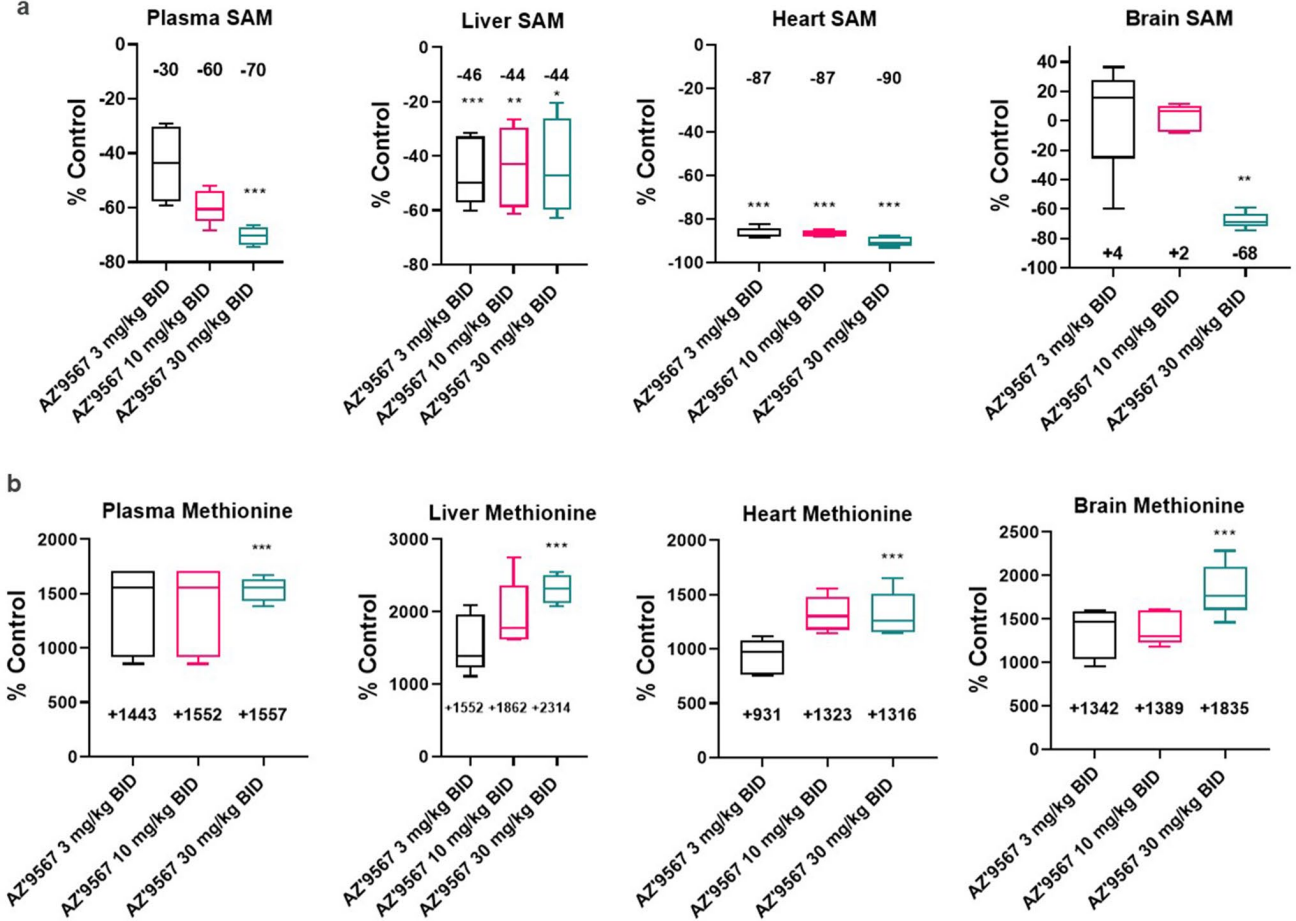
Tissue alterations of SAM and methionine metabolites induced by MAT2A inhibitor. Targeted LC–MS across plasma, liver, brain and heart of treated animals showing % of increase and decrease (relative to control levels), in basal methionine and SAM respectively with dose escalation of AZ’9567. *p < 0.05, **p < 0.01, ***p < 0.001

In vivo target engagement and selectivity of our MAT2A inhibitor were then established by measuring SAM and methionine levels in various organs of treated versus control animals via targeted LC–MS (Fig. 2). As proof of target engagement, plasma SAM decreased to levels 70% lower than the vehicle controls at 30 mg/kg BID with a dose response identified. In brain no significant change was recorded for 3 and 10 mg/kg BID but at 30 mg/kg BID a reduction in SAM of -68% was seen, whilst the heart observations recorded close to a 90% decrease at all dose levels. Of note, in liver, where the MAT1A isoform is prevalently expressed, a very consistent ~ 45% decrease was seen over all doses, suggesting a lack of MAT2A selectivity (Fig. 2). Furthermore, we measured a significant change in basal methionine levels in both plasma and tissues post AZ’9567 treatment at all doses. A maximal change of + 1557% was recorded in plasma with the top dose. In liver, heart and brain tissue maximal increases in the high dose group of + 2314%, + 1316% and 1835% were identified. In the brain, we measured a significant accumulation of methionine also at low doses of the inhibitor, where SAM levels did not decrease. We believe this is due to the systemic circulation and the high concentrations of methionine in the plasma, considering that AZ’9567 has low brain penetrance (Kpu,u = 0.03 ± 0.01) (Atkinson et al. 2024) and methionine uptake across the Blood Brain Barrier has been reported in the literature (Nagata et al. 2011; O'Tuama et al. 1991; Uda et al. 2010; Zaragozá 2020). Furthermore, these findings are in agreement with those obtained with another next-generation MAT2A inhibitor (Li et al. 2022).

### MAT2A inhibition-induced perturbations in the hepatic proteome

To characterize proteomic expression changes in response to AZ’9567 exposure, the liver proteome of control and treated animals was analysed by a label-free quantitative proteomic technology. MAT2Ai treatment induced a strong proteome modulation that was similar across all doses tested (SI Fig. 4a). Overall, an average total profile of 3840 proteins were quantified. With the lowest dose (3 mg/kg BID), we detected around 357 differentially expressed proteins compared to vehicle control (adjusted p < 0.05 and Log2 FC > 0.5 or < −0.5); while for the middle and highest doses there were 467 and 519 differentially expressed proteins, respectively.

**Fig. 3:**
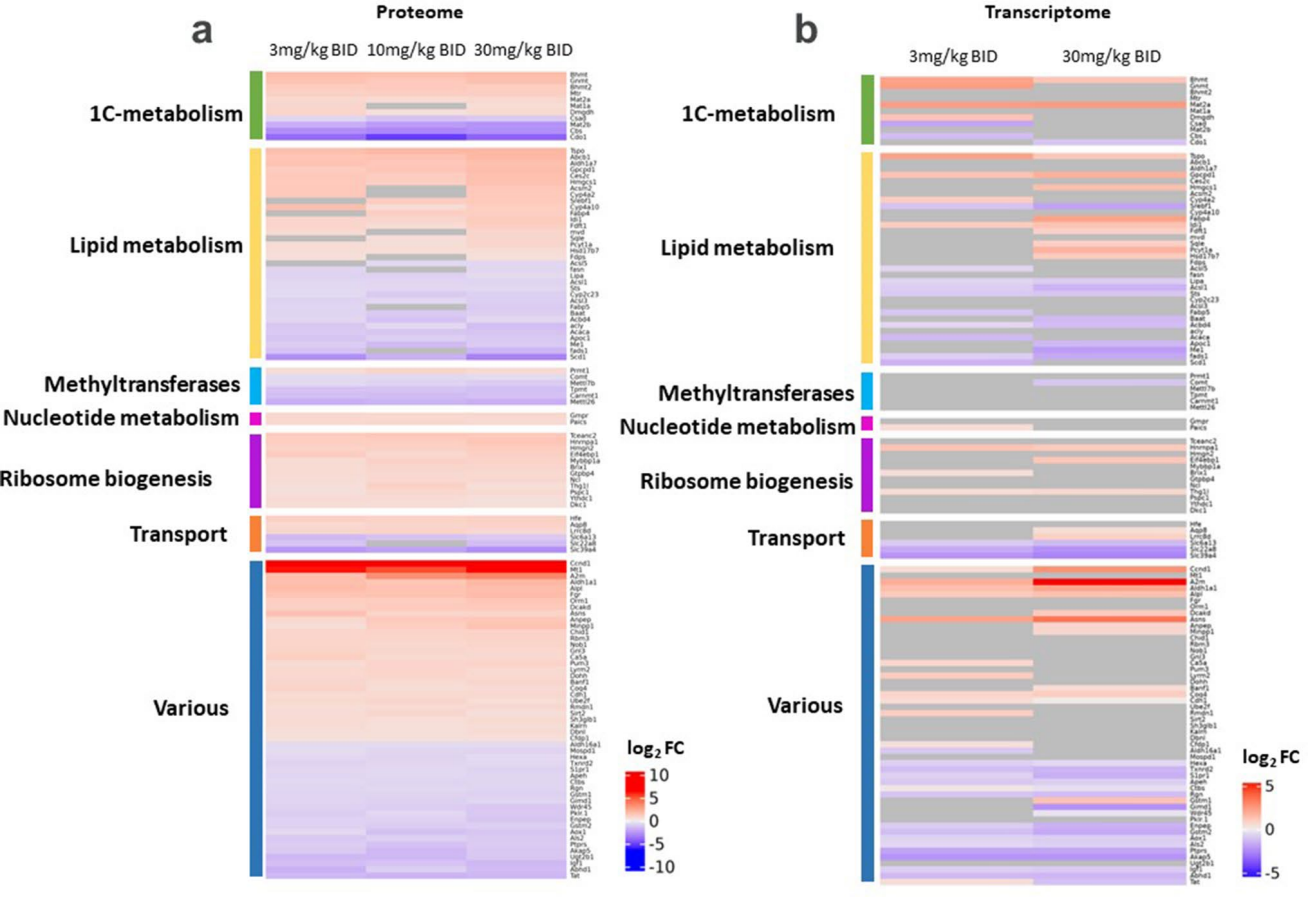
Characterization of the proteome profile induced by MATi and mRNA-protein correlation. **a** Heat map of the most significant protein alterations across MATi-dosed groups. The heat map displays relative fold-change (log_2_FC) in the hepatic protein content across treatments and it shows only hits differentially altered in at least 2 doses with mean absolute Log_2_FC across doses > 0.5. Selected genes were grouped according to biological pathways. **b** gene expression of the proteomic hits significantly altered upon AZ’9567 treatment at 3 mg/kg and 30 mg/kg BID doses. Differentially upregulated and down regulated proteins/mRNAs are indicated in red and blue respectively. Grey boxes indicate proteins/mRNAs that did not pass the significance threshold

In order to obtain an overview of the effects of AZ’9567 treatment on rat liver, the most significant proteins were categorized accordingly to their biological processes and displayed in a heat-map (Fig. 3a and supplementary Table 3). We found that, among 123 selected hits, 33 (27%) were proteins involved in lipid metabolism and 11 (9%) were enzymes belonging to 1C-metabolism. 12 hits (9.7%) were categorized as regulators of transcription and ribosomal biogenesis, while ion transporters and various methyltransferases both represented 4.9% of the most significantly altered proteins. All the remaining hits were involved in miscellaneous cellular processes comprising cell cycle regulation (ie Ccnd1), acute and oxidative stress responses (ie Mt1, Txnrd2, Aox1), glutathione (ie Gstm1, Gstm2), amino acids (ie Asns, Tat) and energy metabolism (ie Pklr.1).

Among the hits belonging to 1-C metabolism, we observed a significant upregulation of key methyltransferases within the methionine cycle, such as betaine-homocysteine S-methyltransferases (BHMT and BHMT2), glycine N-methyltransferase (GNMT) and 5-methyltetrahydrofolate-homocysteine methyltransferase (MTR), together with enhanced levels of the enzyme dimethylglycine dehydrogenase (DMGDH). On the other hand, we detected a significant depletion of cystathionine β-synthase (CBS), the enzyme that catalyses the first step of the trans-sulfuration pathway, and a significant decrease in the levels of two other enzymes involved in trans-sulfuration (cysteine deoxygenase 1 (Cdo1) and cysteine sulfinic acid decarboxylase (CSAD)). Finally, we observed increased levels of both MAT2A and MAT1A, which is likely a compensatory response to MAT2A inhibition, and a significant reduction of MAT2B, the regulatory subunit of MAT2A.

A relevant finding of the proteomic analysis was that MAT2A inhibition altered a significant number of proteins involved in lipid metabolism. Interestingly, of the 33 hits identified, 11 were key enzymes of cholesterol biosynthesis (ie Hmgcs1, Cyp4a2, Idi1, Fdft1, mvd, Sqle, Hsd17b7, Fdps) and one was a transcription factor (Srebf1) involved in the activation of genes related to lipids and cholesterol production. These proteins were all upregulated as well as PCYT1a, GPCPD1 (glycerophosphocholine phosphodiesterase 1) and ABCB1, which are involved in the synthesis, metabolism and transport of the most abundant cytomembrane phospholipid, phosphatidylcholine. Among the remaining hits related to lipids, the majority were enzymes regulating fatty acids metabolism (ie Scd1, Acaca, Fads1, Cyp4a10, Ces2c, Acsl5/3, Me1) and they were mainly downregulated (Fig. 3a and Supplementary Table 3).

To investigate RNA:protein correlation patterns, a parallel transcriptomic analysis was performed in livers from animals treated with the low and high doses of inhibitor. Differential expression was detected in 2080 and 1638 genes respectively (SI Fig. 4b) and alterations at the transcriptional level were confirmed for several of the most significantly altered proteins (Fig. 3b).

### MAT2A inhibition-induced perturbations in the hepatic metabolome and lipidome

**Fig. 4:**
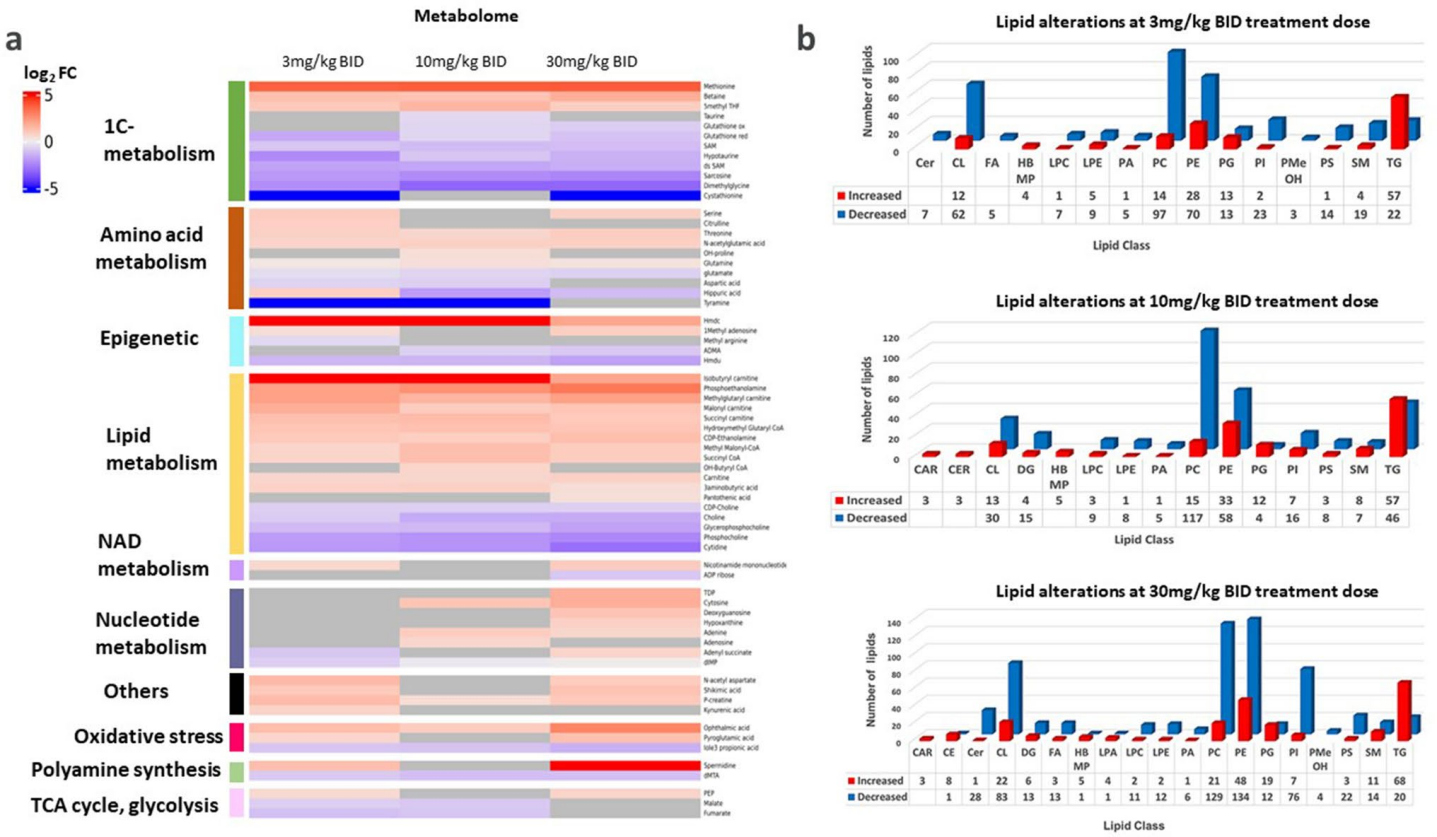
Liver Metabolome and lipidome signatures induced by MATi. **a** heat map of Log_2_ relative fold-change (Log_2_FC) in hepatic 1-C metabolites as quantified by LC–MS. **b** Effect of MATi on the hepatic lipidome. *BA* bile acids, *Cer* ceramide, *CL* cardiolipin, *FA* fatty acid, *PLC* lysophosphatidylcholine, *PLE* lysophosphatidylethanolamine, *LPA* lysophosphatidic acid, *PA* phosphatidic acid, *PC* phosphatidylcholine, *PE* phosphatidylethanolamine, *PS* phosphatidyl serine, *PI* phosphatidylinositol, *PG* phosphoglycerolipid, *TG* triglyceride, *CL* cholesterol ester, *DG* diacylglycerol

A preliminary metabolic assessment via mass spectrometry imaging (MSI) showed how, accumulation of AZ’9567 in the liver, correlated with alterations in the relative abundance of key metabolites of 1C-metabolism (SI Fig. 5). LC–MS-based targeted metabolomics enabled the analysis of more than 300 metabolites belonging to 1C-metabolism and closely related pathways in liver. As indicated in the heat map of Fig. 4a, the inhibitor induced specific metabolic signatures that largely correlated with the altered protein profiles.

Focusing on the effects on 1-C metabolism, we consistently detected a decrease in SAM levels and a parallel large accumulation of methionine in all treatment groups. We also identified a perturbation of polyamine metabolism with a decrease in MTA together with an accumulation of spermidine.

Of note, key metabolites of the methionine and folate cycles and trans-sulfuration either significantly decreased or accumulated. Betaine and 5-methyltetrahydrofolate (5 M-THF) accumulated concomitantly with methionine. On the other hand, biomarkers of the trans-sulfuration pathway, such as cystathionine, hypotaurine, taurine and the anti-oxidant metabolite glutathione (both reduced and oxidised) were significantly reduced. In addition, we observed accumulation of pyroglutamic acid, a marker of glutathione deficiency, and increased levels of ophthalmic acid, a candidate oxidative stress biomarker.

The levels of several by-products of nucleotide metabolism, which is tightly connected to the 1-C metabolic network, were also altered by MAT2A inhibition. Of note, while some intermediates of purine metabolism (hypoxanthine, IMP, adenine, guanosine) were increased, the most striking alteration was a significant and consistent decrease in the levels of cytidine.

Finally, we observed a significant reduction of choline and its related metabolites phosphocholine, CDP-choline and glycerophosphocholine which are involved in the synthesis and catabolism of the lipid phosphatidylcholine (PC). Furthermore, we detected a parallel large increase in phosphoethanolamine, and to a lesser extent also of CDP-ethanolamine, two intermediates in the synthesis of the lipid phosphatidylethanolamine.

**Fig. 5:**
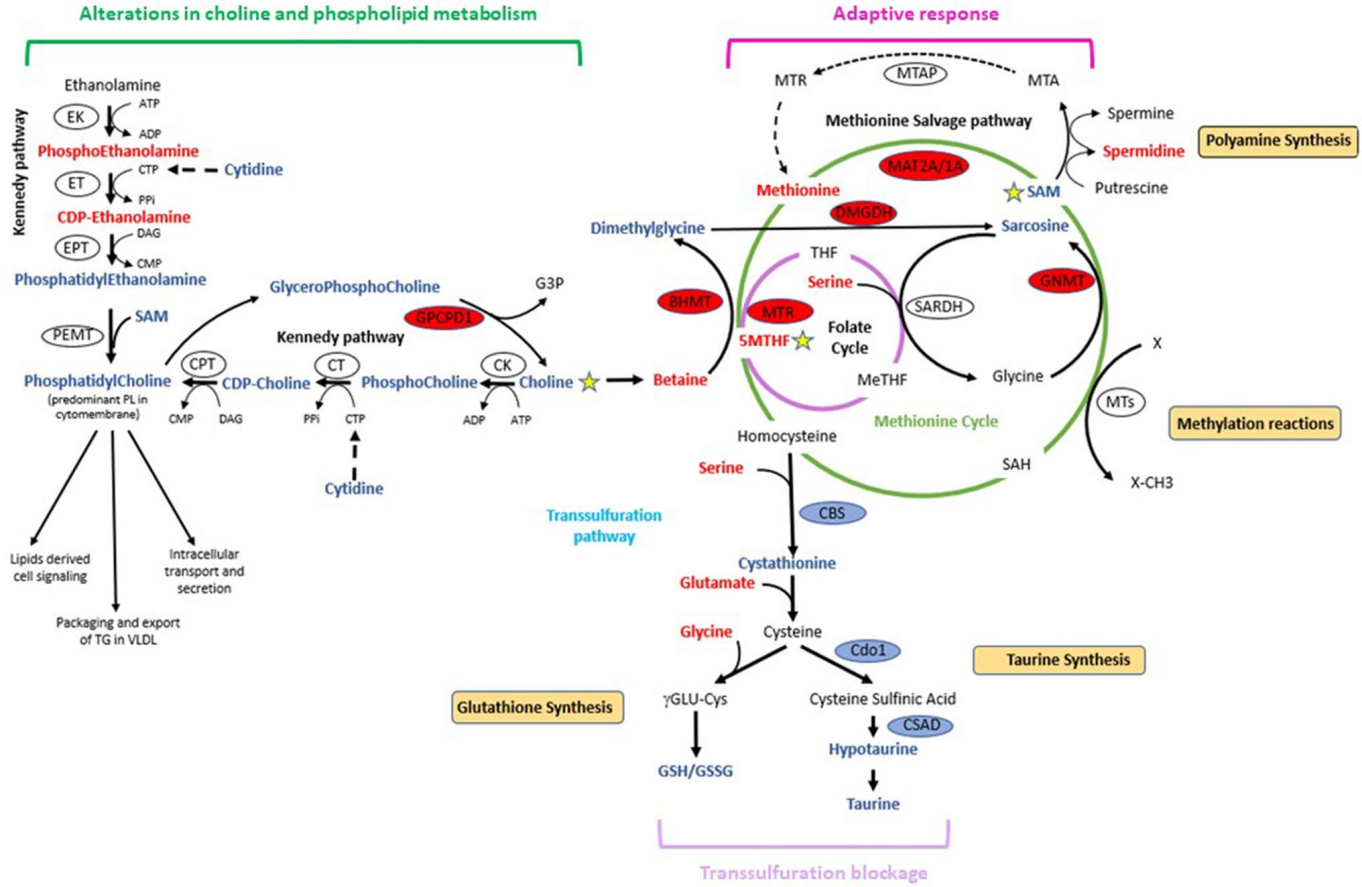
Schematic presentation of changes in hepatic 1-C and choline metabolism following MATi. Observed changes in proteins and metabolites are depicted in red and blue to indicate upregulation and downregulation respectively

Taken together, these data provide support for methionine and SAM being key metabolites for the correct and efficient circulation of 1C units through 1-C metabolism. They provide evidence that MATi inhibition alters the flux of 1-C metabolism and induces specific metabolic profiles that might create vulnerabilities such as altered redox metabolism and phospholipid synthesis and homeostasis (Fig. 5).

Given these metabolic observations and the previously identified differential expression of several proteins involved in cholesterol biosynthesis and fatty acid metabolism, we sought to investigate the impact of MATi treatment on the hepatic lipidome via an untargeted lipidomic analysis. As shown in Fig. 4b, inhibition of MAT activity was associated with hepatic accumulation of triglycerides (TG), cholesteryl esters (CE) and phosphoglycero lipids (PG). There was also a significant decrease in the number of several types of phospholipids, with phosphatidyl cholines, phosphatidyl ethanolamines, phosphatidyl inositols (PI) and cardiolipins (CL) among the most significantly down regulated.

Taken together, our findings indicate that MATi is associated with several changes in lipid/phospholipid profiles that might adversely impact lipid homeostasis.

## Discussion

Exploiting the synthetic lethality between MTAP loss and MAT2A/PRMT5 inhibition could lead to the development of novel precision oncology drugs. However, while the antitumor effect of various MAT2A inhibitors is well documented, there is currently a paucity of studies on the impact of this new class of drugs on non-tumour, healthy cells. We designed this multi-omic study to address this imbalance and provide a pre-clinical, system-wide understanding of MAT2A inhibition in normal tissues.

Given MAT1A is highly and preferentially expressed in the liver, our observation of significant SAM depletion (45% of control value) and perturbation of both MAT1A and MAT2A at a proteomic level suggest we do not have pharmacological selectivity. We have also confirmed this in primary human hepatocyte cultures, that solely express MAT1A, as treatment with our inhibitor led to SAM depletion (data not shown). In addition, designing in selectivity will likely be challenging given the 84% sequence homology of MAT1a and MAT2a and examination of crystal structures at the ligand binding sites show all residues with side-chains oriented towards the ligand are 100% identical.

Tool compounds may have poly-pharmacology and we cannot exclude a contribution of unknown AZ’9567 off-targets to the observed effects. However, we have a robust secondary pharmacology data package which defines the off-target profile. Finally, compelling multi-omic pathway level evidence supports target effects that are MAT1A/MAT2A inhibition driven and more broadly due to perturbations of 1C-metabolism. Similarly to in vivo genetic MAT1A deletion (Lu et al. 2001), pharmacological inhibition of MAT activity reduced hepatic SAM and downstream metabolites involved in polyamine metabolism and the trans-sulfuration pathway. The levels of choline and related metabolites were also greatly reduced. Conversely, MATi led to a dramatic accumulation of methionine and, to a lesser extent, other amino acids including serine and threonine. These metabolic signatures were linked to deregulation at the proteomic level of numerous enzymes involved in 1-C metabolism, cholesterol biosynthesis, fatty acid metabolism, and lipid transport. Overall, our results suggest that systemic depletion of SAM not only affects SAM-dependent cellular function but also results in a dramatic adaptive response, in an attempt to increase SAM, which leads to altered flux of 1-C units through 1-C metabolism. This compensatory mechanism is likely associated with increased risk of oxidative stress, liver steatosis and altered systemic lipid homeostasis.

Central to the observed adaptive response to SAM depletion is the regeneration of methionine from homocysteine to maintain cellular methylation potential. This is achieved through reduced CBS activity, which leads to downregulation of the trans-sulfuration pathway, and is accompanied by increases of BHMT and MTR, in order to enhance homocysteine remethylation capacity (Fig. 5). In this context, betaine, a product of choline degradation (Craig 2004), and 5MTHF, an intermediate metabolite of the folate cycle, are the methyl donors. As part of this adaptive response, accumulation of GNMT modulates SAM utilization and drives the flux of methyl groups towards methionine regeneration. However, although hepatic MAT1A and MAT2A expression is up-regulated at the protein level after MAT2A inhibition, the capacity of the liver of treated animals to metabolize methionine is markedly diminished, which leads to hypermethioninemia and subsequent reduced SAM levels. Our observations of increased MAT2A expression after AZ’9567, are in line with the literature as MAT2A is known to compensate for MAT1A loss. MAT1A, which maintains the differentiated state of hepatocytes, is downregulated in multiple liver diseases, during de-differentiation and in hepatocellular carcinoma (Avila et al. 2000; Cai et al. 1996; Lee et al. 2004). Conversely, MAT2A is transcriptionally induced in human hepatocellular carcinoma, during liver growth and de-differentiation (Huang et al. 1998; Martínez-Chantar et al. 2003; Yang et al. 2008).

The shunt of 1-C metabolism away from the trans-sulfuration pathway resulted in a significant reduction of biomarkers of the trans-sulfuration pathway (glutathione, hypotaurine, taurine). Inadequate levels of the antioxidant glutathione impair the cellular redox potential posing a risk of oxidative stress. In agreement with this, we detected enhanced levels of pyroglutamic and ophthalmic acids, biomarkers of hepatic glutathione depletion and oxidative stress (Dello et al. 2013; Gamarra et al. 2019; Soga et al. 2006). Critically, we have characterised the metabolism of AZ’9567 in both in vitro (primary human hepatocytes) and in vivo studies (rat plasma) and ruled out any contribution from glutathione-related metabolism of AZ’9567.

An important consequence of enhanced utilization of betaine to support BHMT-dependent re-methylation of homocysteine, is the depletion of choline and the associated metabolic sequalae. Choline is an essential nutrient: apart from being a source of methyl groups, it is also a precursor of membrane phospholipids (PL), lipoproteins and acetylcholine (Ueland 2011). Choline is primarily obtained from the diet or from the conversion of phosphatidylethanolamine (PE) to PC followed by catabolism to choline (Fagone and Jackowski 2013). Although it can be oxidized in the liver and kidney to betaine, it is predominantly shunted into the synthesis of PC via the CDP-choline pathway (the choline branch of the so-called ‘Kennedy pathway’ for phospholipid synthesis). Additionally, around 30% of hepatic PC is generated by phosphatidylethanolamine methyltransferase (PEMT) from PE (Gibellini and Smith 2010). This pathway involves the methylation of PE with SAM being the methyl donor. Therefore, MAT inhibitor-dependent reductions in SAM and 1-C metabolism perturbations are likely to affect both PC biosynthetic pathways. In agreement with this, we observed significantly decreased levels of choline, and alterations in the metabolites of both branches of the Kennedy pathway (phosphocholine, CDP-choline, phosphoethanolamine, CDP-ethanolamine). Of note, these alterations might be linked to the marked decrease we observed in cytidine. This pyrimidine, in fact, is a precursor of cytidine triphosphate (CTP) needed in the PC and PE biosynthetic pathways for the generation of CDP-choline and CDP-ethanolamine intermediates (Fig. 5). We also observed decreased amounts of glycerophosphocholine, which we infer is due to the increased levels of GPCPD1, a key enzyme in the generation of choline via hydrolysis of the lipid PC (Stewart et al. 2012). This suggests cells respond to MAT2A inhibition by redirecting choline from phospholipid catabolism towards maintenance of homocysteine methylation. In agreement with this, the untargeted lipidomic analysis underlined a marked decrease in PC and related phospholipids. Overall, these data are indicative of a cellular response that favours methionine regeneration at the expense of PC production and phospholipid homeostasis.

PC is a main component of VLDL particles and biosynthesis, and turnover of PC is important for the formation of these particles and export of lipids from hepatocytes to tissues through the blood (Jacobs et al. 2004; Nishimaki-Mogami et al. 2002; Yao and Vance 1988). When this pathway is disturbed, lipid droplets accumulate in hepatocytes causing steatosis (Li and Vance 2008; Yao and Vance 1988). Notably, studies performed in MAT1A knockout mice indicate that MAT1A supports lipoprotein homeostasis and ensures adequate VLDL assembly and secretion (Cano et al. 2011). Together with our data showing that MAT inhibition impairs PC synthesis, these observations suggest that MATi might reduce VLDL secretion and thereby engender hepatic lipid accumulation. In support of this hypothesis, our lipidomic analysis evidenced an increase in triglycerides species.

Interestingly, we also observed a significant downregulation in CL, a mitochondrial specific phospholipid that is mainly located in the inner mitochondrial membrane. The implications of this alteration will require further investigation that is beyond the scope of this study. However, it is worth mentioning that CL is crucial for the integrity of the respiratory chain complex (Houtkooper and Vaz 2008). This suggests that changes in CL, and other PL profiles, could affect mitochondrial membrane structure–function relationships and ultimately have adverse effects on liver mitochondrial oxidative energy metabolism.

Impairments in liver PC metabolism and dysfunctions of the pathways involved in de novo lipid synthesis and catabolism are key in the pathogenesis of non-alcoholic fatty liver disease (NAFLD) (Friedman et al. 2018; Masoodi et al. 2021; Postic and Girard 2008). This condition is characterized by the accumulation of hepatic fat and presents a wide spectrum of hepatic metabolic disorders ranging from simple steatosis to inflammatory steatohepatitis (NASH) and cirrhosis. We show here that, in addition to altering PC levels, MATi affected several proteins involved in both lipid metabolism and the progression of NAFLD (Table 3 supplementary data). Our proteomic studies evidenced significant alterations in the levels of enzymes involved in fatty acid metabolism (SCD, ACACA, FADS1, CYP4A10, CES2C, ACSL4) and lipid transport (ABCB1, TSPO). Of particular interest is the profound suppression of stearoyl coenzyme A desaturase-1 (SCD1). This protein is a major lipid-metabolising enzyme that catalyses the formation of monounsaturated fatty acids (MUFA-primarily oleate and palmitoleate) from saturated fatty acids (SFA-stearate and palmitate) (Flowers and Ntambi 2008). MUFA are incorporated into complex lipids including triglycerides, membrane phospholipids, and cholesteryl esters, and as constituents of cell membranes they play a crucial role in maintaining membrane fluidity and therefore physiological functions (Paton and Ntambi 2009). The mechanism by which SCD-1 is downregulated in MATi-treated animals, as well as the downstream consequences, are currently unclear. Interestingly, however, mice fed a methionine- and choline-free diet develop hepatic steatosis but also exhibit robust suppression of hepatic SCD-1 expression (Rizki et al. 2006).

Of note, by proteomic analysis we identified accumulation of enzymes belonging to cholesterol biosynthetic pathways (HMGCS1, IDI, FDFT1, SQLE, CYP51A1, HSD17B7, MVD). In agreement with this, the analysis of metabolomic data highlighted accumulation of hydroxymethyl glutaryl CoA (HMG-CoA), an intermediate of cholesterol synthesis produced by the HMG-CoA synthase (HMGCS1), and the lipidomic analysis confirmed an increase in cholesterol esters. The molecular mechanism leading to these alterations remains to be investigated.

The alterations in protein, metabolite and PL profiles in our multi-omic studies are similar to those observed in MAT1A KO mice (Alonso et al. 2017; Lu et al. 2001; Martínez-Chantar et al. 2002). However, there is no histopathological evidence of liver steatosis in MAT2Ai-treated mice, which contrasts with observations in MAT1A KO mice (Lu et al. 2001). This discrepancy may be due to temporal differences: our toxicological study was relatively short-term (8-days), whereas the effects of MAT1A KO are measured over 8 months. Of note, 3 months old MAT1A KO mice did not exhibit altered hepatic lipid content or major liver damage, although at this age they were more susceptible to choline-deficient diet-induced fatty liver (Cano et al. 2011; Lu et al. 2001). Furthermore, alterations in lipid and carbohydrate metabolism in MAT1A KO mice precede any histological sign of steatosis or NASH (Martínez-Chantar et al. 2002). Therefore, it is possible that MATi-induced liver injury may be observed in preclinical models at a later stage or after prolonged treatment schedules. It is also possible that other factors could delay the manifestation of damage: the choline content in the diet, for example, might be important in determining the susceptibility of MATi treated animals to liver injury. Within the time frame of the in vivo studies, we identified dose-dependent microscopic changes in the pancreas consisting of significant atrophy of the exocrine parenchyma. These lesions were observed only in toxicological rat studies but not in efficacy studies in tumor-bearing mice. Furthermore, an in vivo study with an additional MAT2A inhibitor did not show pancreatic toxicity at equivalent target cover (data not shown). The literature evidence does not describe pancreatic toxicity following MAT1A KO despite a drop of 80% of pancreatic SAM. SAM depletion has been linked to acute pancreatitis in the context of a choline deficient and ethionine supplemented diet (CDE) in young mice (Lu et al. 2003). However, experimental conditions such as sex, age and diet have been reported to influence the susceptibility of the animals to CDE-induced pancreatitis and induce a variety of lesions to the pancreas (Lu et al. 2003). The acute pancreatic damage consisted of extensive necrosis, edema and acute inflammatory infiltration, but we observed pancreatic lesions that did not involve edema nor inflammatory infiltrations. Hence, while more studies are needed to understand the mechanism of this toxicity and the role of SAM and MAT genes in the pancreas, we do not think that the exocrine pancreatic changes have impacted the multi-omic results in the liver. Rather, we believe that our liver findings are primarily driven by on-target pharmacology of both MAT1A and MAT2A inhibition.

## Final conclusions

This study serves as an example of mechanistic discovery safety and validates the application of multi-omics tools to systematically understand the biological pathways and molecular features involved in response to drugs. Furthermore, this integrative approach offers the opportunity to identify biomarkers of early toxicity and ultimately define translational strategies to improve assessment of therapeutic safety in clinical studies.

### Supplementary Information

Below is the link to the electronic supplementary material.

Supplementary file1 (DOCX 4996 KB)

## Data Availability

Data is available from the corresponding author on reasonable request.
