# Supplementary material for: Mechanistic safety assessment via multi-omic characterisation of systemic pathway perturbations following in vivo MAT2A inhibition
Source: Arch Toxicol. 2024 May 17;98(8):2589–603. doi: 10.1007/s00204-024-03771-w (PMC11272821; doi:10.1007/s00204-024-03771-w)
Supplement: Supplementary file 1 — Supplementary file1 (DOCX 4996 KB) [file 204_2024_3771_MOESM1_ESM.docx]

**Supplementary information**

**Mechanistic safety assessment via multi-omic characterisation of systemic pathway perturbations following in vivo MAT2A inhibition.**

Valentina Fogal^1,15^, Filippos Michopoulos^2^, Andrew Jarnuczak^3^, Ghaith M. Hamza^4^, Stephanie Harlfinger^5^, Paul Davey^6^, Heather Hulme^7^, Stephen J Atkinson^6^, Piotr Gabrowski^8^, Tony Cheung^9^, Michael Grondine^9^, Clare Hoover^10^, Jonathan Rose^11^, Chandler Bray^15^, Alison J Foster^12^, Sean Askin^13^, Muntasir Mamun Majumder^14^, Paul Fitzpatrick^14^, Eric Miele^4^, Ruth MacDonald^11^, Hector Keun^15^, Muireann Coen^1^

^1^Oncology Safety, Clinical Pharmacology & Safety Sciences, R&D, AstraZeneca, Cambridge, UK

^2^Bioscience, Research and Early Development, Oncology R&D, AstraZeneca, Cambridge, UK

^3^Data Sciences & Quantitative Biology, Discovery Sciences, R&D, AstraZeneca, Cambridge, UK

^4^Discovery Biology, Discovery Sciences, R&D, AstraZeneca, Boston, USA

^5^DMPK, Oncology R&D, AstraZeneca, Cambridge, UK

^6^Chemistry, Oncology TDE AstraZeneca, Cambridge, UK

^7^Imaging and Data Analytics, Clinical Pharmacology and Safety Sciences, AstraZeneca R&D, Cambridge, UK

^8^Owkin, Paris, France

^9^Oncology R&D, AstraZeneca, R&D Boston, USA

^10^Oncology Safety Pathology, Clinical Pharmacology & Safety Sciences, R&D, AstraZeneca, Waltham, USA

^11^Animal Science & Technologies, R&D, AstraZeneca, Cambridge, UK

^12^Regulatory Toxicology and Safety Pharmacology, Clinical Pharmacology & Safety Sciences, R&D, AstraZeneca, Cambridge, UK

^13^Advanced Drug Delivery, Pharmaceutical Sci, R&D, AstraZeneca, Cambridge, UK

^14^Respiratory and Immunology Safety Science, Safety Science and Discovery RIA, Gothenburg, Sweden

^15^Cancer Metabolism & Systems Toxicology Group, Division of Cancer, Department of Surgery and Cancer, Imperial College London, London, UK

Corresponding author: muireann.coen@astrazeneca.com

**Supplementary figures**

**SI Fig.1 Secondary Pharmacology Profile.** Activity (a defined IC_50_ or EC_50_ value) was detected for AZ’9567 at 33 secondary pharmacology targets (receptors, ion channels, transporters and enzyme) however none of these targets was covered by the plasma free Cmax observed in rats following BID dosing of 3, 10 or 30 mg/kg for 7 days (dotted lines)


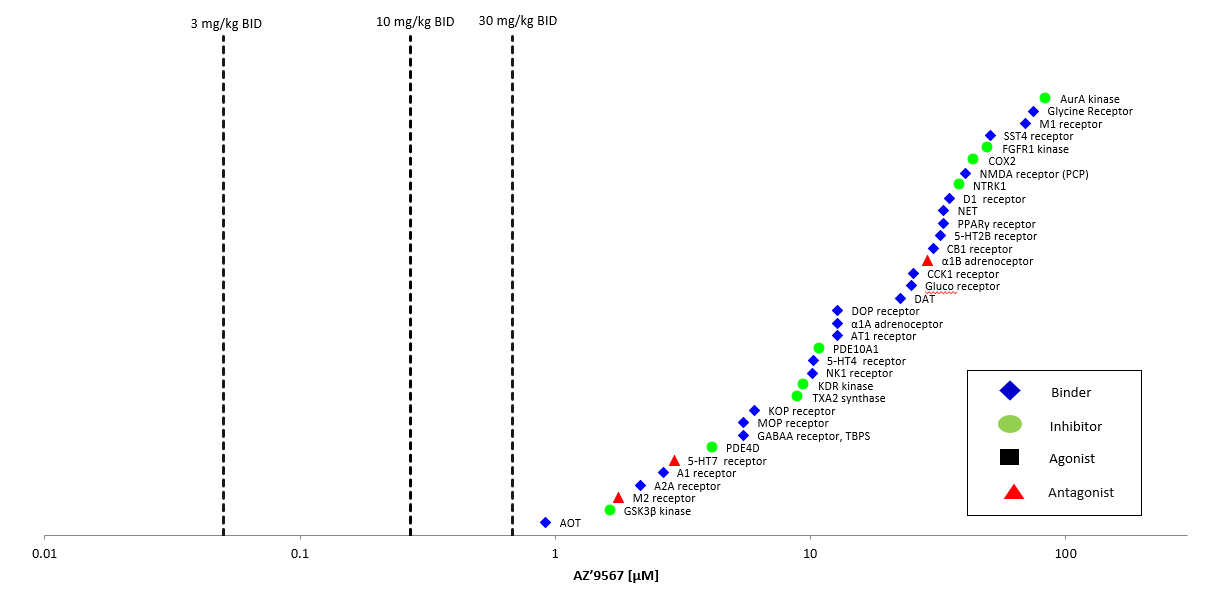


**SI Fig.2 Correlation between body weight/food consumption and treatment induced changes on liver metabolome and proteome. (a)** Food consumption and body weight (BW) changes (% relative to day 1) in treated and control (Gp) animals. **(b,c)** Volcano plots visualizing the results of bodyweight regression analysis. In the proteomics data **(b)**, 113 proteins (2.9%) showed association with BW change, while 559 proteins (14%) showed an association with Treatment. In metabolomics **(c)**, 12 metabolites (3.9%) showed association with BW change and 36 metabolites (12%) showed an association with Treatment. The x-axis is the coefficient value (effect size) for either “BW percent change” or “Treatment” extracted from the linear model and y-axis is its log10 transformed p-value. Each point represents a protein/metabolite.

**a**

**Day**

**1**

**2**

**3**

**4**

**5**

**6**

**7**

**8**

**-10**

**0**

**10**

**% Change Bodyweight**

**(relative to Day-1)**

Control Gp1

3 mg/kg

Control Gp3

10 mg/kg

Control Gp5

30 mg/kg

**Day**


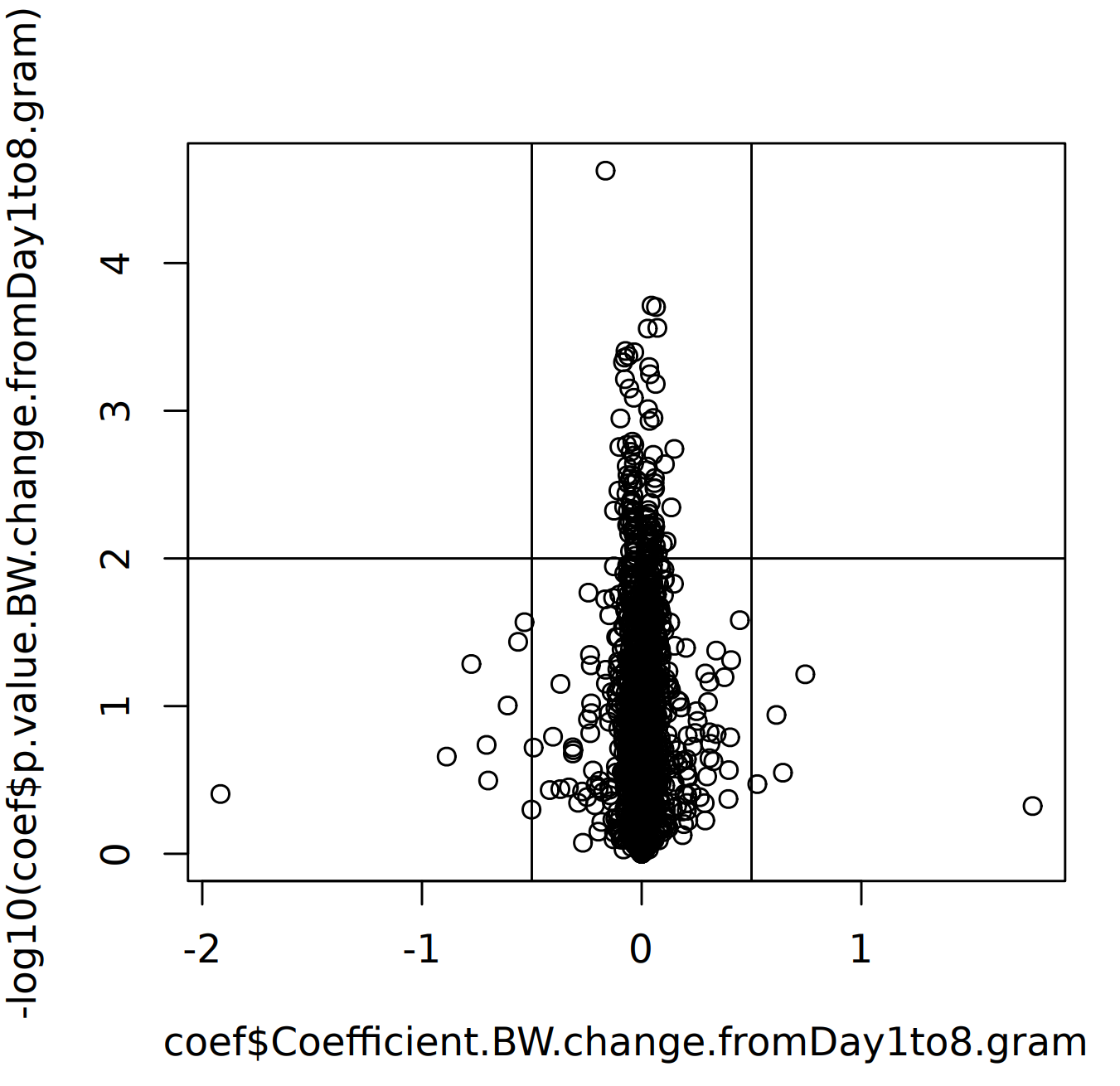

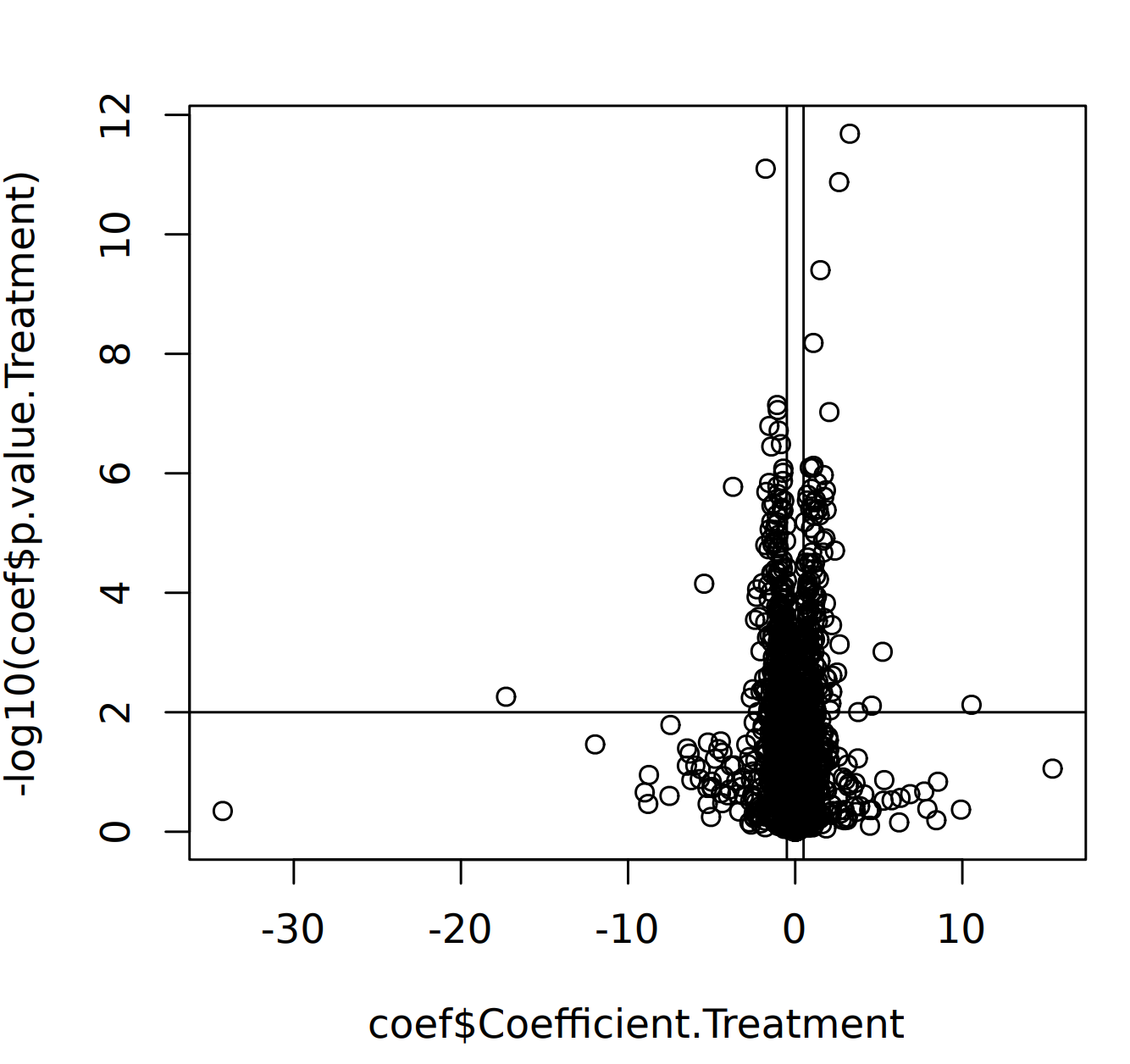


**Body weight association blot**

**Treatment association blot**

**b**


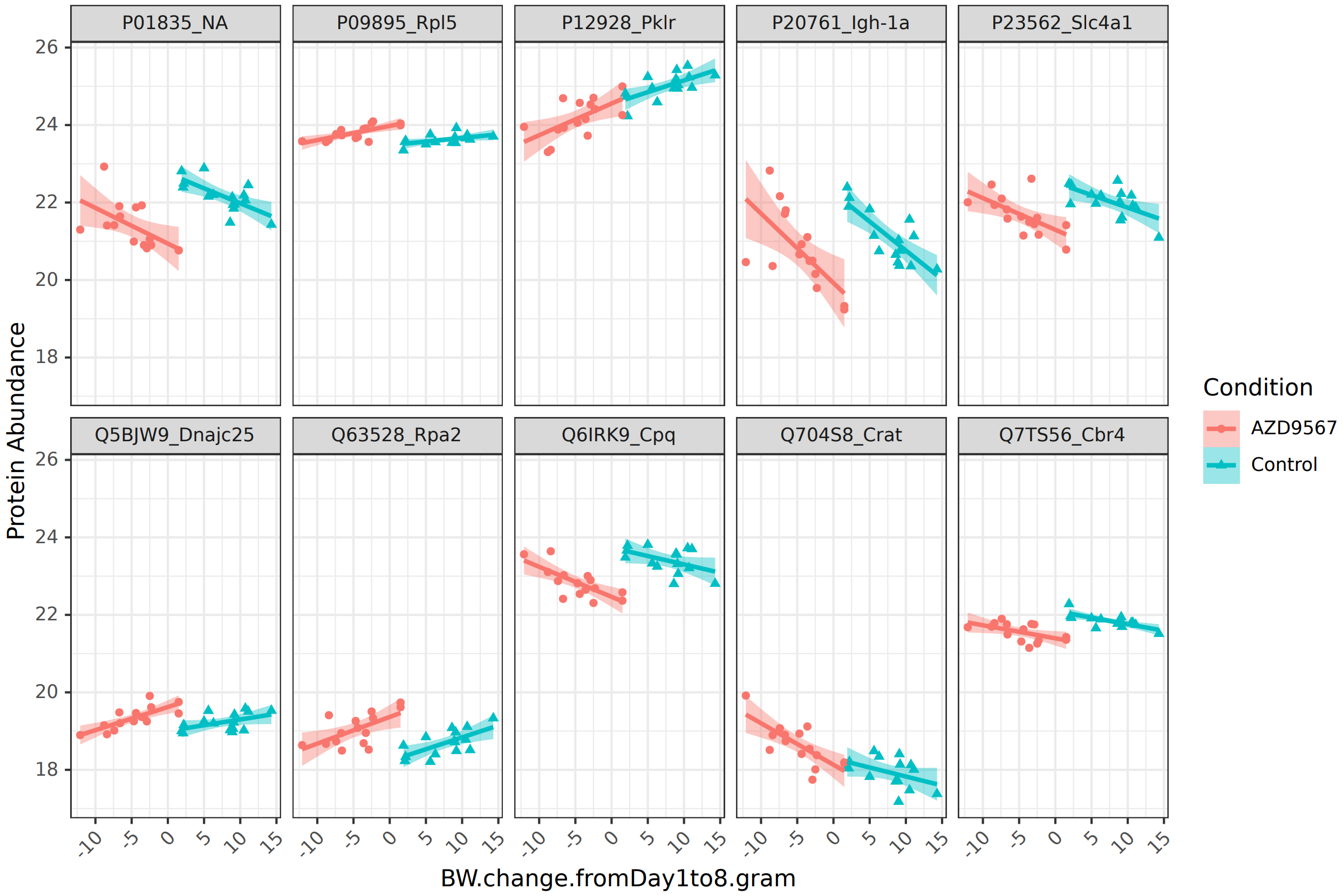

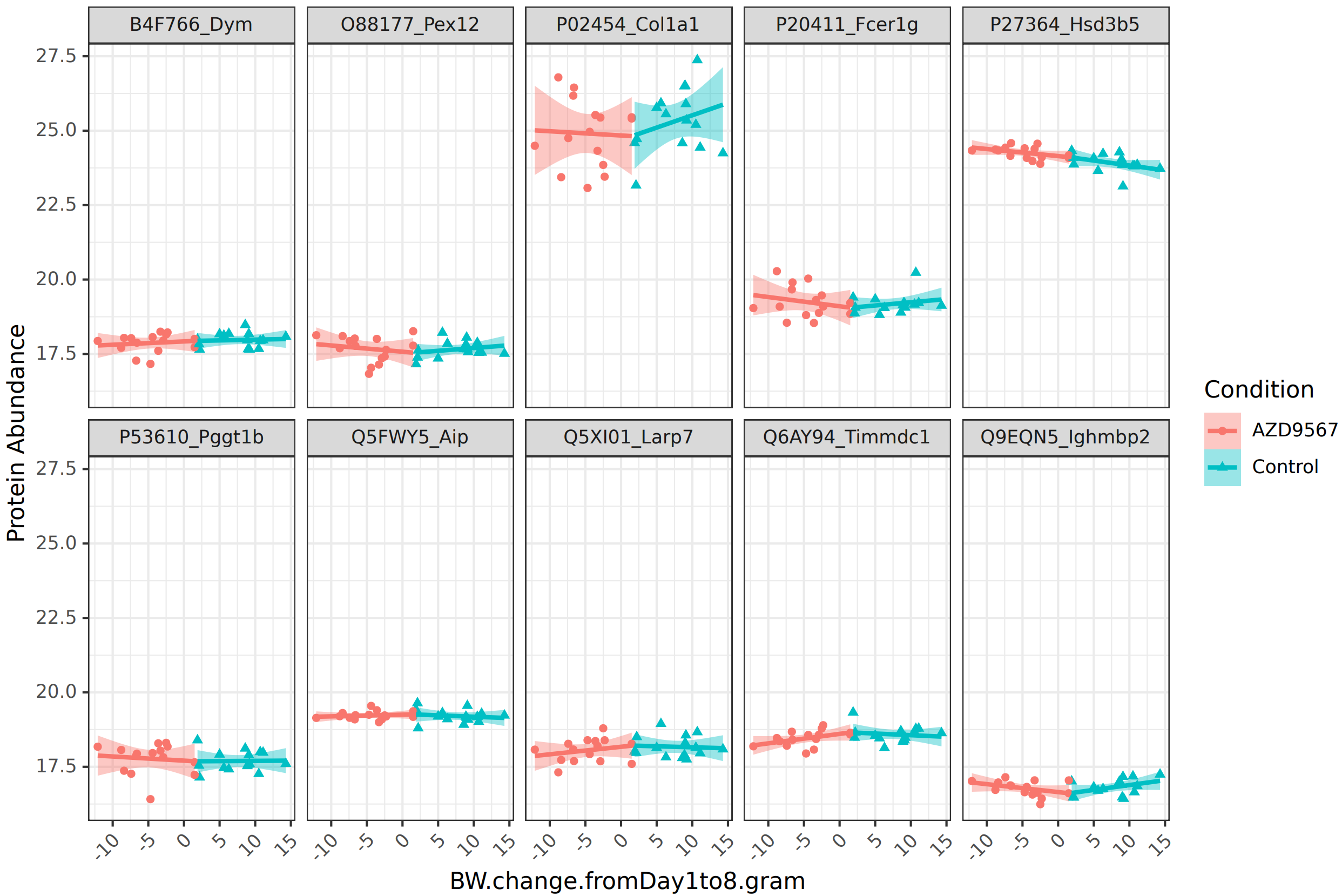


**Top proteins with strongest association with BW**

**BW vs protein abundance for top proteins with largest coefficient for treatment**

**c**


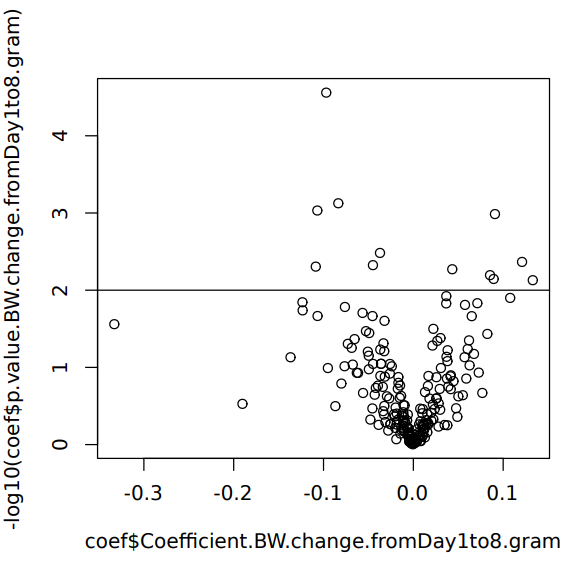


**Body weight association blot**


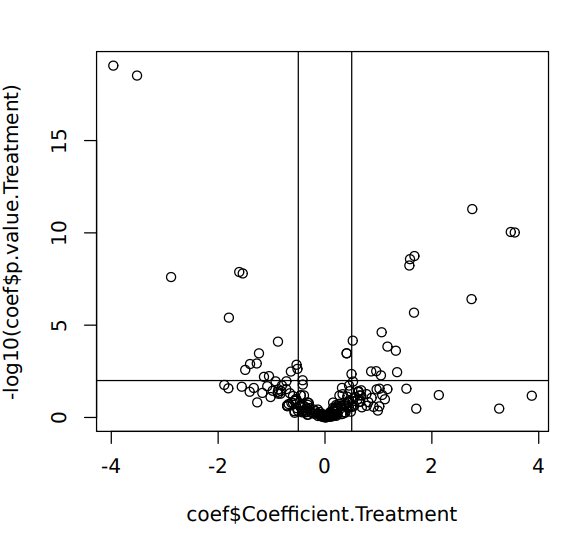

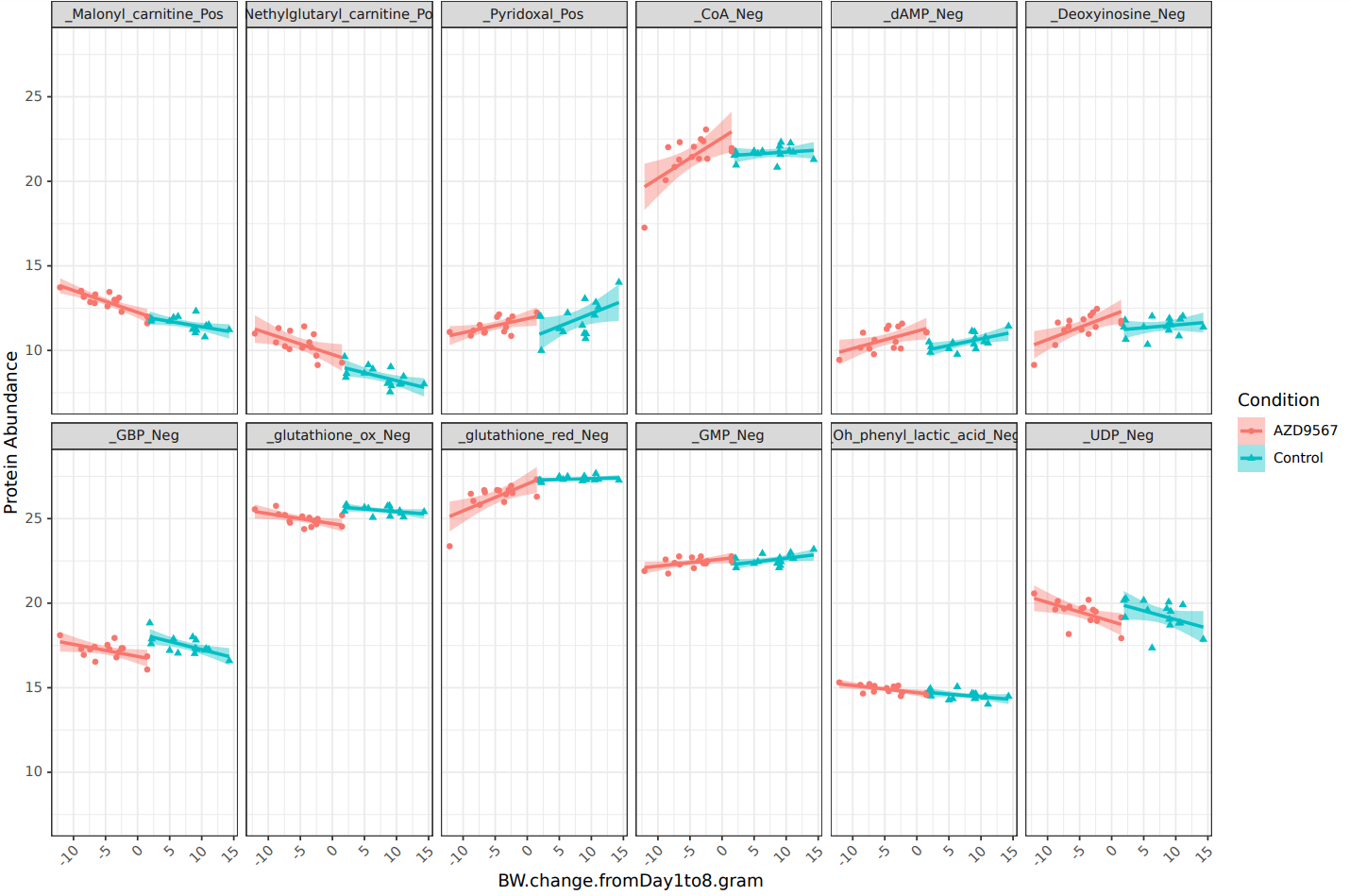


**Metabolites associated with BW**

**SI Fig.3 Histopathology. (a)** Liver pathology. No significant abnormalities detected in liver at any dose group. At 30 mg/kg of AZMAT2Ai there is a decrease in glycogen stores consistent with decreased food intake. **(b)** clinical chemistry. **
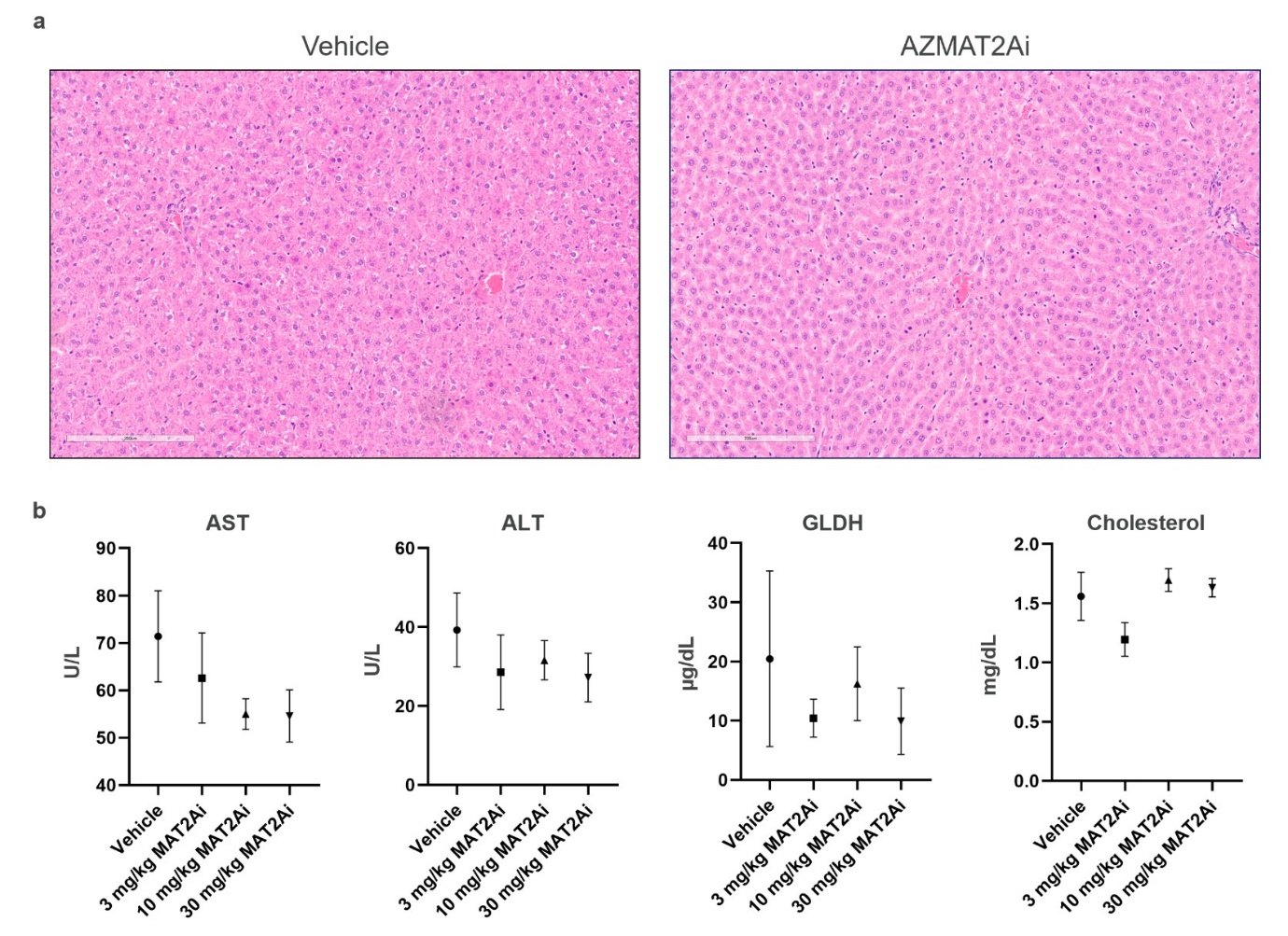
**

**SI Fig.4** Volcano plots showing proteomics (**a**) and transcriptomic (**b**) data from animals treated with increased doses of AZ’9567. Dots represent identified proteins/mRNAs. A selection of hits among those that display both large magnitude fold change (x axis) and high statistical significance (-log10 of p value, y axis) are indicated. To identify differentially modulated proteins/mRNAs, the Log2 fold changes relative to control were filtered by adj p-value less than 0.05, and only values >0.5 and <-0.5 were considered in order to select for upregulated and downregulated proteins/mRNAs respectively. Dashed horizontal line shows the p values cut off, and the two vertical dashed lines indicate down/up regulated proteins.

**
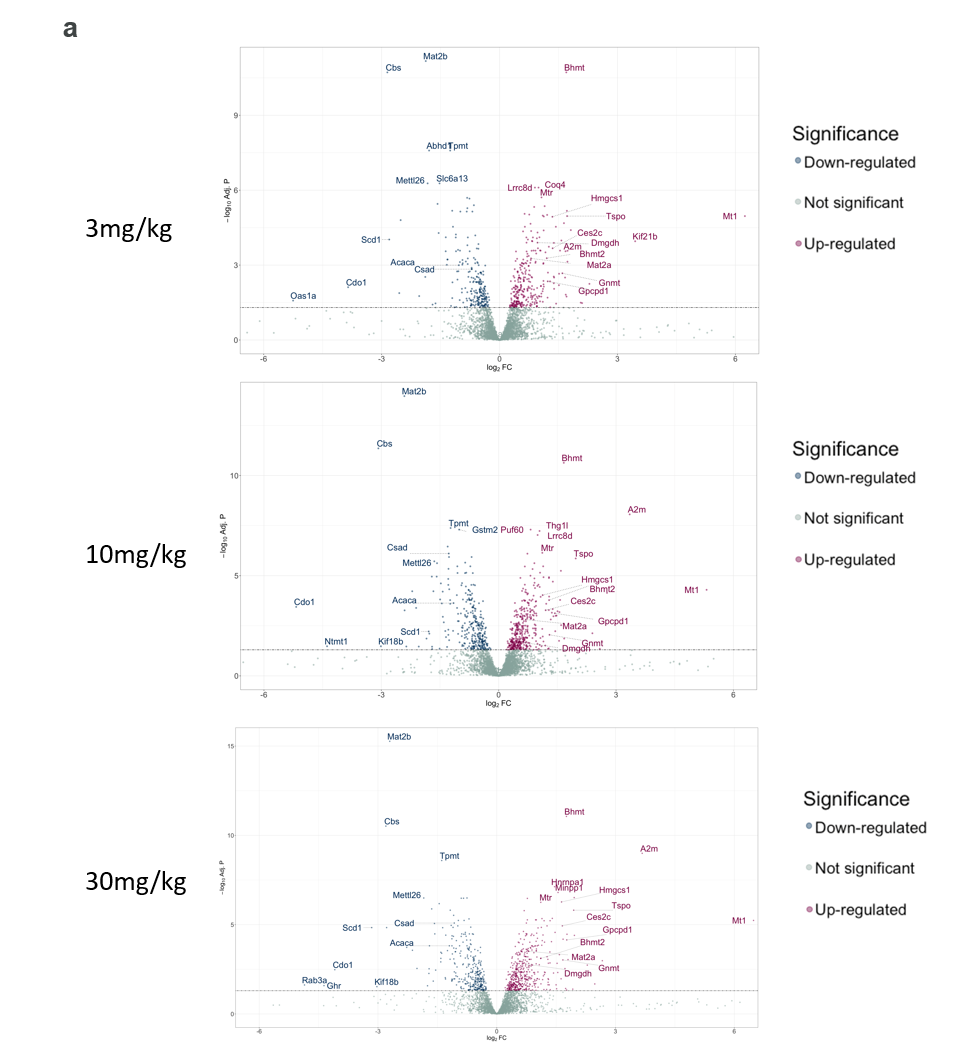
**

**
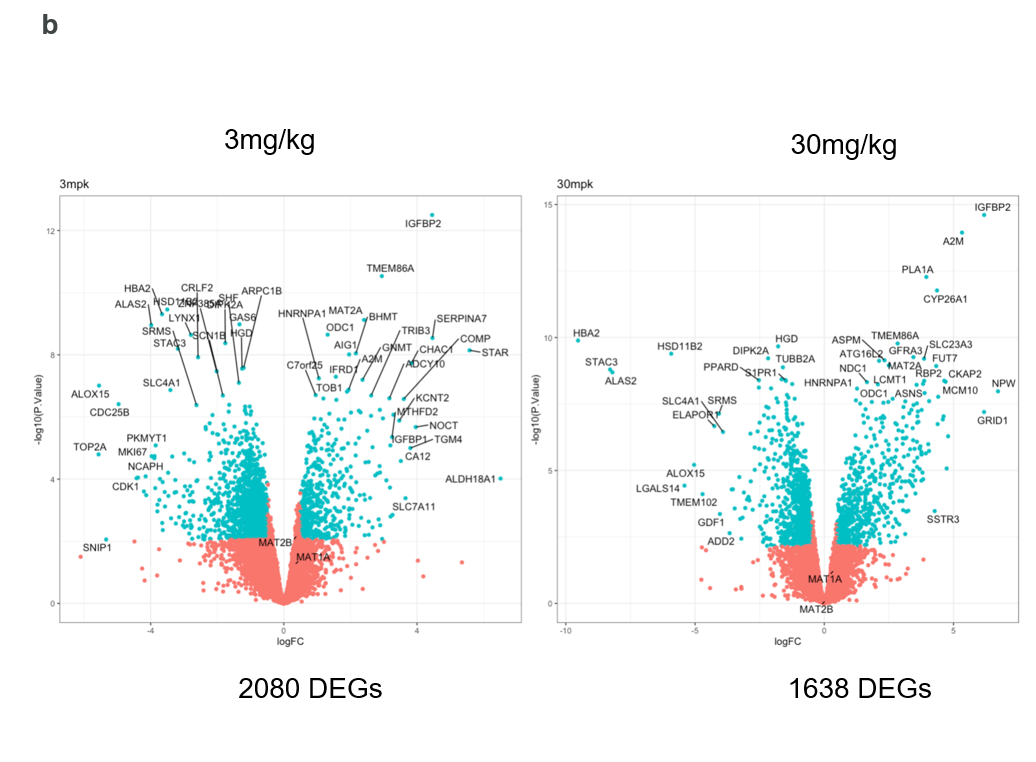
**

**SI Fig.5 Mass spectrometry imaging of livers from anti MAT2A treated animals.** (**a**) Upper panel: liver accumulation and relative quantification of AZ’9567. The images are representative of liver sections showing distribution of the compound. The compound is distributed mostly homogenously across the liver with hotspots of increased abundance in the blood vessels. The graph below shows relative quantification of the compound in the different AZMAT2Ai dose group. Points show biological replicates. Error bars show standard deviation. (**b**) Heat map representing the relative abundance of metabolites of 1-C metabolism as detected in anti MAT2A treated and vehicle livers. Each column corresponds to a different animal. Colours represent the z score (deviation of relative abundance from mean for each metabolite) in each individual animal. Asterisks show results of kruskal-wallis test * p-value <0.05, **** p-value <0.0001


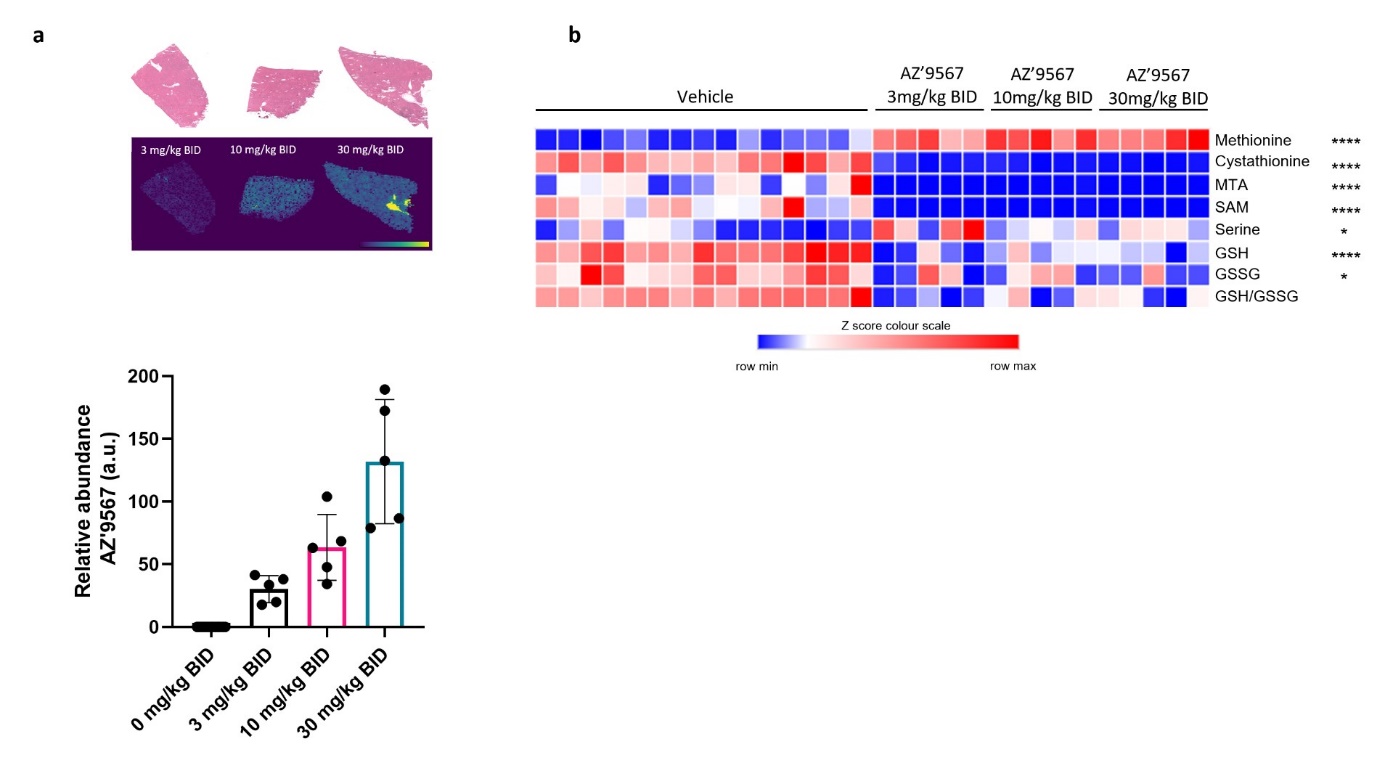


**SI Fig. 6 Volcano plots of fused positive and negative polar metabolite data sets.** Volcano plots generated for each dose level after comparison to vehicle control group. (
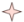
) Statistically significant metabolites with QC CV<30%, |log(2) Fold Change|>0.5 and p-value< 0.05, (
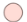
) metabolite with p-value< 0.05 and (
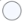
) metabolites with p-value > 0.05

**
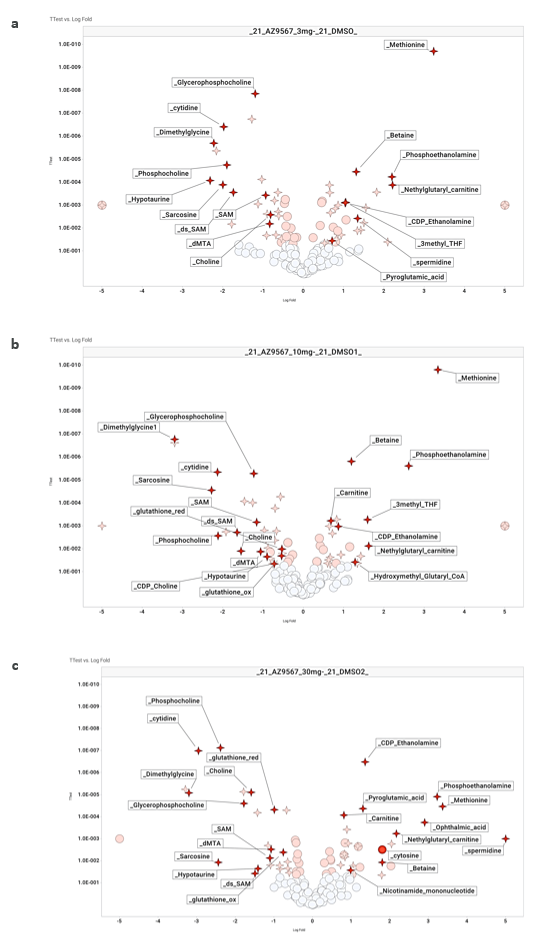
**

**Table S1. Effect of AZ’9567 in *in vitro* radioligand binding, enzyme and functional assays**

| Target^a^ | Binding K_i_ or Enzyme IC_50_ (μM) | Functional IC_50_ or EC_50_ (μM) | Pharmacological mode of action |
| --- | --- | --- | --- |
| Adenosine Transporter (binding guinea pig, functional human) | 0.9 | 1.4 | Antagonist |
| Glycogen synthase kinase-3 β | 1.6 | NT | ND |
| M_2_ muscarinic receptor | 1.8 | 17.1 | Antagonist |
| A2A adenosine receptor | 2.2 | >100 | ND |
| A_1_ adenosine receptor | 2.6 | >30.1 | ND |
| 5-HT_7_ receptor | 2.9 | 2.9 | Antagonist |
| Phosphodiesterase PDE4D2 | 4.1 | NT | ND |
| GABAA receptor, TBPS site (rat) | 5.5 | NT | ND |
| μ opioid receptor | 5.5 | NT | ND |
| κ opioid receptor | 6.0 | NT | ND |
| Thromboxane A2 synthase | 9.0 | NT | ND |
| Kinase insert domain receptor kinase (KDR) | 9.5 | NT | ND |
| NK_1_ neurokinin receptor | 10.2 | NT | ND |
| 5-HT_4_ receptor | 10.3 | NT | ND |
| Phosphodiesterase PDE10A2 | 10.8 | NT | ND |
| AT_1_ angiotensin receptor | 12.7 | NT | ND |
| α_1A_ adrenoceptor | 12.7 | NT | ND |
| δ opioid Receptor | 12.8 | NT | ND |
| Dopamine transporter (DAT) | 22.6 | NT | ND |
| Glucocorticoid Receptor NR3C1 | 24.8 | NT | ND |
| CCK1 cholecystokinin receptor | 25.3 | NT | ND |
| α_1B_ adrenoceptor | 28.9 | 28.9 | Antagonist |
| CB_1_ cannabinoid receptor | 30.3 | >100 | ND |
| 5-HT_2B_ receptor | 32.4 | NT | ND |
| Peroxisome proliferator-activated receptor-γ | 33.3 | NT | ND |
| Noradrenaline Transporter (NET) | 33.3 | NT | ND |
| D_1_ dopamine receptor | 35.0 | NT | ND |
| Neurotrophic receptor kinase 1 (NRK1) | 38.3 | NT | ND |
| NMDA receptor (Phencyclidine site; rat) | 40.6 | NT | ND |
| Prostaglandin-endoperoxide synthase 2 (COX2) | 44.0 | NT | ND |
| Fibroblast growth factor receptor 1 kinase (FGFR1 kinase) | 49.9 | NT | ND |
| SST_4_ somatostatin receptor | 50.6 | NT | ND |
| M_1_ muscarinic receptor | 69.7 | NT | ND |
| Glycine receptor, Strychnine-Sensitive (rat | 75.0 | NT | ND |
| Aurora A kinase | 84.1 | NT | ND |

^a^ all human except where noted.

NT not tested

ND not determined or insufficient data to define pharmacological mode of action

**Table S2 AZ’9567 In vitro safety profile covering cardiac, hepatic, mitochondrial and cytotoxicity risk.**

| **Safety Assay (IC50s μM)** | **AZ’9567** |
| --- | --- |
| hERG | 33.09 |
| NaV1.5 | >40.02 |
| IKs | >33.33 |
| Kv4.3 | >40.02 |
| CaV-L (VER) | >100 |
| CaV-L (DIL) | >100 |
| Hep G2 Glu/Gal Ratio | NV |
| Hep G2 Glu | >100 |
| Hep G2 Gal | >100 |
| THP-1 Cytotox | >50 |
| HepG2 Spheroid Cytotox | 58.72 |

Full curve IC_50_ (μM) for hERG (human ether-a-go-go-related gene) and the cardiac ion channel panel (sodium and potassium ion channels; Nav1.5, IKs, Kv4.3), along with the L-type Calcium channel (Cav-1.2 Ver; verapamil, DIL; diltiazem), HepG2 C3A 3D spheroids, HepG2 C3A (2D cultures in glucose or galactose media), and THP1 cytotoxicity.

**Table S3. Selection of differentially expressed proteins in rat liver following AZ’9567 treatment**

| **Gene name** | **Protein name** | **Function** | **Change** | **Log2FC** | | |
| --- | --- | --- | --- | --- | --- | --- |
|  |  |  |  | **3mg/kg** | **10mg/kg** | **30mg/kg** |
| **1-C METABOLISM** | | | | | | |
| **Bhmt** | Betaine homocysteine methyl transferase | Methionine and homocysteine metabolism | ↑ | 1.7 | 1.7 | 1.8 |
| **Gnmt** | Glycine N-methyl transferase | Methionine and homocysteine metabolism | ↑ | 1.6 | 1.3 | 1.7 |
| **Bhmt2** | Betaine homocysteine methyl transferase 2 | Methionine and homocysteine metabolism | ↑ | 1.2 | 1.3 | 1.1 |
| **Mtr** | 5-Methyl tetrahydrofolate methyltransferase | Methionine and homocysteine metabolism | ↑ | 1.1 | 1.1 | 1.1 |
| **Mat2a** | Methionine adenosyl transferase 2A | Methionine metabolism, SAM production | ↑ | 0.8 | 0.7 | 0.8 |
| **Mat1a** | Methionine adenosyl transferase 1A | Methionine metabolism, SAM production | ↑ | 0.9 | - | 0.6 |
| **Dmgdh** | Dimethyl glycine dehydrogenase | Choline catabolism, methionine metabolism | ↑ | 1 | 0.5 | 0.7 |
| **Csad** | Cysteine sulfinic acid decarboxylase | Transsulfuration, taurine synthesis | ↓ | -0.7 | -1.3 | -1.1 |
| **Mat2b** | Methionine adenosyl transferase 2B | Regulatory subunit of MAT2A | ↓ | -1.9 | -2.4 | -2.7 |
| **Cbs** | Cystathionine beta-synthase | Transsulfuration, cysteine synthesis | ↓ | -2.9 | -3.1 | -2.8 |
| **Cdo1** | Cysteine dioxygenase type 1 | Transsulfuration, cysteine oxydation | ↓ | -3.9 | -5.2 | -4.1 |
| **LIPID METABOLISM** | | | | | | |
| **Tspo** | Translocator protein | Cholesterol transport across mitochondria membrane (C) | **↑** | 1.7 | 2 | 1.9 |
| **Abcb1** | ATP binding cassette subfamily B member 1 | Phospholipids translocation across membrane (PL) | **↑** | 1.4 | 1.6 | 2 |
| **Aldh1a7** | Aldehyde dehydrogenase family 1 subfamily 7 | Oxidoreductase | **↑** | 1.5 | 1.4 | 1.7 |
| **Gpcpd1** | Glycerophosphocholine phosphodiesterase | Glycerophospholipids catabolism (PL) | **↑** | 1.2 | 1.4 | 1.8 |
| **Ces2c** | Carboxyl esterase 2C | Glycerolipids and phospholipids metabolism (GL and PL) | **↑** | 1.4 | 1.2 | 1.7 |
| **Hmgcs1** | Hydroxy-methyl-glutaryl-CoA synthase 1 | Cholesterol synthesis (C) | **↑** | 1.3 | 1.1 | 1.6 |
| **Acsm2** | Acyl CoA synthetase medium chain family 2A | Fatty acids activation (FA) | **↑** | 1.4 | - | 1.4 |
| **Cyp4a2** | Cytochrome P450 family 4 subfamily A member 2 | Cholesterol synthesis (C) | **↑** | 1.4 | - | 1.3 |
| **Srebf1** | Sterol regulatory element binding factor 1 | Activation of genes involved in Cholesterol synthesis (C) | **↑** | - | 1.2 | 1.3 |
| **Cyp4a10** | Cytochrome P450 family 4 subfamily A polipeptide 10 | Fatty acids metabolism (FA) | **↑** | 1.7 | 0.7 | 1.1 |
| **Fabp4** | Fatty acids binding protein 4 | Fatty acids uptake, transport and metabolism (FA) | **↑** | - | 1.1 | 1.1 |
| **Idi1** | Isopentenyl-diphosphate delta isomerase 1 | Cholesterol synthesis (C) | **↑** | 0.9 | 0.9 | 1.3 |
| **Fdft1** | Farnesyl-diphosphate farnesyltransferase 1 | Cholesterol synthesis (C) | **↑** | 0.9 | 0.7 | 1.1 |
| **mvd** | Mevalonate diphosphate decarboxylase | Cholesterol synthesis (C) | **↑** | 0.7 | - | 0.9 |
| **Sqle** | Squalene epoxidase | Cholesterol synthesis (C) | **↑** | - | 0.6 | 0.8 |
| **Pcyt1a** | Phosphate cytidylytransferase 1, choline alpha | Phosphatidylcholine biosynthesis (PL) | **↑** | 0.7 | 0.6 | 0.8 |
| **Hsd17b7** | Hydroxysteroid 17-beta dehydrogenase 7 | Cholesterol synthesis (C) | **↑** | 0.6 | 0.6 | 0.6 |
| **Fdps** | Farnesyl diphosphate synthase | Cholesterol synthesis (C) | **↑** | 0.5 | - | 0.5 |
| **Acsl5** | Acyl-CoA synthetase long chain family member 5 | Fatty acids synthesis and degradation (FA) | **↓** | - | -0.6 | -0.6 |
| **Fasn** | Fatty acids synthase | Fatty acids biosynthesis | **↓** | -0.7 | - | -0.7 |
| **Lipa** | Lipase A | Lipid degradation (C, TG) | **↓** | -0.8 | -0.8 | -0.5 |
| **Acsl1** | Acyl-CoA synthetase long chain family member 1 | Fatty acids degradation (FA) | **↓** | -0.7 | -0.7 | -0.8 |
| **Sts** | Steroid sulfatase | Regulation and formation of steroids | **↓** | -0.7 | -0.7 | -0.6 |
| **Cyp2c23** | Cytochrome P450 family 2 subfamily C polipeotide23 | Fatty acids metabolism (FA) | **↓** | -0.6 | -1.1 | -0.9 |
| **Acsl3** | Acyl-CoA synthetase long chain family member 3 | Fatty acids synthesis and degradation (FA) | **↓** | -0.8 | -0.6 | -0.9 |
| **Fabp5** | Fatty acids binding protein 5 | Fatty acids uptake and transport | **↓** | -0.7 | - | -1.0 |
| **Baat** | Bile acid-CoA:amino acid N-acyltransferase | Fatty acids metabolism | **↓** | -0.8 | -1.1 | -0.9 |
| **Acbd4** | Acyl-CoA binding domain containing 4 | Acyl-Coa dependent lipid metabolism | **↓** | -0.7 | -1.3 | -0.6 |
| **Acly** | ATP citrate lyase | Synthesis of Acetyl-CoA | **↓** | -1.1 | -0.6 | -0.9 |
| **Acaca** | Acetyl-CoA carboxylase alpha | Fatty acids synthesis (FA) | **↓** | -1 | -1.2 | -1.2 |
| **Apoc1** | Apolipoprotein C1 | Lipid transport, HDL and VLDL metabolism | **↓** | -1.2 | -0.9 | -1.1 |
| **Me1** | Malic enzyme 1 | Fatty acids biosynthesis (FA) | **↓** | -1 | -1.5 | -1.0 |
| **Fads1** | Fatty acids desaturase 1 | Biosynthesis of highly unsaturated fatty acids (HUFA) (FA) | **↓** | -1.3 | - | -1.6 |
| **Scd1** | Stearoyl-CoA desaturase | Biosynthesis of mono unsaturated fatty acids (MUFA) (FA) | **↓** | -2.8 | -1.8 | -3.2 |
|  |  | METHYLTRANSFERASES |  |  |  |  |
| **Prmt1** | Protein arginine methyltrasferase 1 | Methylation of arginine residues | **↑** | 0.6 | 0.9 | 0.7 |
| **Comt** | Catechol-O-methyltransferase | Degradation of catecholamine transmitters | **↓** | -0.5 | -0.6 | -0.6 |
| **Mettl7b** | Methyltransferase like 7B | Transfer to methyl group to hydrogen sulfite | **↓** | -0.6 | -0.8 | -1.0 |
| **Tpmt** | Thiopurine S-methyltransferase | Metabolism of thiopurine drugs | **↓** | -1.3 | -1.2 | -1.4 |
| **Carnmt1** | Carnosine N-methyltransferase 1 | Conversion of carnosine to anserine | **↓** | -1.4 | -1.5 | -1.3 |
| **Mettl26** | Methyltransferase like 26 |  | **↓** | -1.8 | -1.6 | -1.8 |
|  |  | NUCLEOTIDES METABOLISM |  |  |  |  |
| **Gmpr** | Guanosine monophosphate reductase | Purine metabolism, deamination of GMP to IMP | **↑** | 0.8 | 0.7 | 0.8 |
| **Paics** | Phosphoribosylaminoimidazole carboxylase and Phosphoribosylaminoimidazolesuccinocarboxamide syntase | Purine biosynthesis | **↑** | 0.7 | 0.8 | 0.7 |
|  |  | **TRANSCRIPTIONAL REGULATION/RIBOSOME BIOGENESIS** |  |  |  |  |
| **Tceanc2** | Transcription elongation factor A N-terminal and central domain containing 2 | Regulation of transcriptional elongation | **↑** | 1.2 | 1.4 | 1.3 |
| **Hnrnpa1** | Heterogeneous nuclear ribonucleoprotein A1 | mRNA processing, splicing and transport | **↑** | 1.1 | 1.1 | 1.5 |
| **Hmgn2** | High mobility group nucleosomal binding domain 2 | Maintenance of transcriptionally active chromatin | **↑** | 1.0 | 1.0 | 1.2 |
| **Eif4ebp1** | Eukaryotic translation initiation factor 4E binding protein 1 | Regulation of translation | **↑** | 1.1 | 0.8 | 1.0 |
| **Mybbp1a** | MYB binding protein 1a | Regulation of transcription | **↑** | 0.7 | 1.1 | 1.0 |
| **Brix1** | Biogenesis of ribosomes BRX1 | Ribosome biogenesis | **↑** | 0.7 | 0.9 | 0.9 |
| **Gtpbp4** | GTP binding protein 4 | Ribosome biogenesis | **↑** | 0.7 | 0.8 | 0.8 |
| **Ncl** | Nucleolin | Synthesis and maturation of ribosomes | **↑** | 0.6 | 0.8 | 0.8 |
| **Thg1l** | TRNA-histidine guanylyltransferase 1 like | tRNA processing, mitochondrial dynamics and bioenergetic function | **↑** | 0.5 | 1 | 0.6 |
| **Pspc1** | Paraspeckle component 1 | Regulation of transcription | **↑** | 0.5 | 0.8 | 0.7 |
| **Ythdc1** | YTH domain containing 1 | nRNA processing, mRNA splicing, regulation of MAT2A transcripts expression | **↑** | 0.7 | 0.7 | 0.5 |
| **Dkc1** | Dyskerin pseudouridine synthase 1 | Ribosome biogenesis, rRNA processing | **↑** | 0.6 | 0.6 | 0.6 |
|  |  | **TRANSPORT** |  |  |  |  |
| **Hfe** | Homeostatic ion regulator | Regulation of ion absorption | **↑** | 1.1 | 1.0 | 1.0 |
| **Aqp8** | Aquaporin 8 | Water channel | **↑** | 0.8 | 0.9 | 1.0 |
| **Lrrc8d** | Leucine rich repeat containing 8 VRAC subunt D | Ion channel | **↑** | 0.9 | 1.0 | 0.8 |
| **Slc6a13** | Solute carrier family 6 member 13 | Transport of GABA, beta alanine, taurine and hypotaurine | **↑** | -1.5 | -1.3 | -1.0 |
| **Slc22a8** | Solute carrier family 22 member 8 | Detoxification, ion transport | **↑** | -1.2 | - | -1.4 |
| **Slc39a4** | Solute carrier family 39 member 4 | Cellular zinc transport and homeostasis | **↑** | -2.5 | -2.2 | -2.8 |
|  |  | **VARIOUS FUNCTIONS** |  |  |  |  |
| **Ccnd1** | Cyclin D1 | Cell cycle, cell division | **↑** | 12.4 | 17.1 | 16.3 |
| **Mt1** | Metallothionein | Anti-oxidant, protect against hydroxyl free radicals | **↑** | 6.2 | 5.3 | 6.5 |
| **A2m** | Alpha-2-macroglobulin | Protease inhibitor | **↑** | 1.7 | 3.3 | 3.7 |
| **Aldh1a1** | Aldehyde dehydrogenase 1 family member A1 | Oxidoreductase involved in retinal metabolism | **↑** | 1.7 | 1.5 | 2.0 |
| **Alpl** | Alkaline phosphatase | Unknown | **↑** | 1.8 | 1.6 | 1.8 |
| **Fgr** | FGR proto-oncogene | Protein tyrosine kinase | **↑** | 1.5 | 1.6 | 1.8 |
| **Orm1** | Orosomucoid 1 | Acute phase reactant, carrier of basic and neutrally charged lipophilic compounds | **↑** | 1.1 | 1.2 | 1.5 |
| **Dcakd** | Dephospho-CoA kinase domain containing | Possibly involved in coenzyme A biosynthetic process | **↑** | 1.0 | 1.3 | 1.3 |
| **Asns** | Asparagine synthetase | Asparagine biosynthesis | **↑** | 1.6 | 0.9 | 1.0 |
| **Anpep** | Alanyl aminopeptidase | Peptides digestion | **↑** | 0.8 | 1.3 | 1.4 |
| **Minpp1** | Multiple inositol-polyphosphate phosphatase 1 | Hydrolase | **↑** | 0.6 | 1.1 | 1.5 |
| **Chid1** | Chitinase domain containing 1 | Inflammatory response | **↑** | 0.9 | 1.0 | 1.0 |
| **Rbm3** | RNA binding motif protein 3 | Stress response | **↑** | 0.9 | 1.0 | 1.0 |
| **Nob1** | NIN1 (RPN12) binding protein 1 homolog | Endonuclease with possible role in mRNA degradation | **↑** | 0.9 | 0.8 | 0.9 |
| **Gnl3** | G protein nucleolar 3 | Stem cells proliferation | **↑** | 1.0 | 0.9 | 0.8 |
| **Ca5a** | Carbonic anhydrase 5A | Ureagenesis and gluconeogenesis | **↑** | 1.1 | 0.7 | 0.9 |
| **Pum3** | Pumilio RNA binding family member 3 | Inhibitor the poly(ADP-ribosyl)ation activity of PARP | **↑** | 0.7 | 0.9 | 1.0 |
| **Lyrm2** | Leucine-tyrosine-arginine motif containing 2 | Regulation of mitochondrial Complex 1 activity | **↑** | 0.8 | 0.9 | 0.7 |
| **Dohh** | Deoxyhypusine hydroxylase | Hypusine biosynthesis | **↑** | 0.8 | 0.9 | 0.7 |
| **Banf1** | BAF nuclear assembly factor 1 | Mitotic nuclear reassembly, chromatin organization | **↑** | 0.8 | 0.7 | 0.9 |
| **Coq4** | Coenzyme Q4 | Ubiquinone biosynthesis | **↑** | 1.0 | 0.6 | 0.7 |
| **Cdh1** | Cadherin 1 | Cell adhesion | **↑** | 0.7 | 0.7 | 0.8 |
| **Ube2f** | Ubiquitin conjugating enzyme E2 F | Ubiquitin conjugation pathway | **↑** | 0.7 | 0.9 | 0.6 |
| **Rmdn1** | Regulator of microtubule dynamics 1 | Regulation of mitosis | **↑** | 0.6 | 0.7 | 0.8 |
| **Sirt2** | Sirtuin 2 | Pleiotropic functions on cellular context | **↑** | 0.7 | 0.9 | 0.5 |
| **Sh3glb1** | SH3 domain containing GRB2 like, endophlin B1 | Maintenance of mitochondria morphology and regulation of apoptotic signaling pathways | **↑** | 0.7 | 0.6 | 0.8 |
| **Kalrn** | Kalirin RhoGEF kinase | Activation of specific Rho GTPase family members | **↑** | 0.7 | 0.6 | 0.6 |
| **Dbnl** | Drebrin like | Receptor mediated endocytosis | **↑** | 0.6 | 0.5 | 0.7 |
| **Cfdp1** | Craniofacial development protein 1 | Maintenance of higher order chromatin organization | **↑** | 0.5 | 0.5 | 0.5 |
| **Aldh16a1** | Aldehyde dehydrogenase 16 family member A1 |  | **↓** | -0.6 | -0.5 | -0.5 |
| **Mospd1** | Motile sperm domain containing 1 | Differentiation and/or proliferation of mesenchymal stem cells | **↓** | -0.5 | -0.7 | -0.7 |
| **Hexa** | Hexosaminidase subunit alpha | Degradation of molecules containing terminal N-acetyl hexosamines | **↓** | -0.6 | -0.7 | -0.6 |
| **Txnrd2** | Thioredoxin reductase 2 | Control of reactive oxygen species levels and regulation of mitochondrial redox homeostasis | **↓** | -0.8 | -0.7 | -0.6 |
| **S1pr1** | Sphingosine-1-phosphate receptor 1 | Differentiation of endothelial cells | **↓** | -0.7 | -0.6 | -0.8 |
| **Apeh** | Acylaminoacyl-peptide hydrolase | Destroying oxidatively damaged proteins | **↓** | -0.7 | -0.7 | -0.7 |
| **Ctbs** | Chitobiase | Degradation of asparagine linked oligosaccharides on glycoproteins | **↓** | -0.6 | -0.7 | -0.7 |
| **Rgn** | Regucalcin | Calcium homeostasis | **↓** | -0.7 | -0.6 | -0.7 |
| **Gstm1** | Glutathione S-transferase mu 1 | Conjugation of reduced glutathione to exogenous and endogenous hydrophobic electrophiles | **↓** | -0.8 | -0.7 | -0.8 |
| **Gimd1** | GIMAP family P-loop NTPase domain containing 1 |  | **↓** | -0.7 | -0.9 | -0.7 |
| **Wdr45** | WD repeat domain 45 | Autophagy | **↓** | -0.8 | -0.9 | -1.1 |
| **Pklr.1** | Pyruvate kinase L/R | Glycolysis | **↓** | -1 | -0.7 | -1.1 |
| **Enpep** | Glutamyl aminopeptidase | Degradation of vasoconstricting angiotensin II/regulation of blood pressure | **↓** | -0.8 | -0.8 | -1.2 |
| **Gstm2** | Glutathione S-transferase mu 2 | Conjugation of reduced glutathione to exogenous and endogenous hydrophobic electrophiles | **↓** | -0.8 | -1.0 | -0.9 |
| **Aox1** | Aldehyde oxidase 1 | Regulation of reactive oxygen species homeostasis | **↓** | -0.6 | -1.3 | -1.2 |
| **Als2** | Alsin Rho guanine nucleotide exchange factor ALS2 | Guanine nucleotide exchange factor for the small GTPase RAB5 | **↓** | -1.1 | -0.9 | -1.1 |
| **Ptprs** | Protein tyrosine phosphate receptor type S | Cell signaling | **↓** | -0.9 | -1.3 | -1.0 |
| **Akap5** | A-kinase anchoring protein 5 | Multivalent scaffold protein | **↓** | -1.0 | -1.6 | -1.1 |
| **Ugt2b1** | UDP glucoronosyltransferase family 2 member B1 | Conjugation and elimination of potentially toxic xenobiotics and endogenous compounds | **↓** | -1.6 | -1.6 | -1.1 |
| **Igf1** | Insulin like growth factor 1 | Growth factor | **↓** | -1.4 | -1.2 | -1.6 |
| **Abhd1** | Abhydrolase domain containing 1 | Serine Esterase | **↓** | -1.8 | -0.9 | -1.5 |
| **Tat** | Tyrosine aminotransferase | Tyrosine breakdown | **↓** | -1.5 | -1.7 | -1.6 |

The change of protein expression level was expressed as Log2 Treated/Control ratio. A value >0.5 represents upregulation whereas a value < -0.5 represents downregulation. P<0.05

(C): Cholesterol, (FA): Fatty acids, (PL): Phospholipids, (TG): Triglycerides, (GL): Glycerolipids

**Supplementary Material & Methods**

**Bodyweight change analysis.** To assess the effects of bodyweight (BW) change versus compound treatment on protein and metabolite expression levels the percentage bodyweight from day 1 to day 8 was calculated. lm() function in R was used to construct a linear model where the dependent variable was the protein/metabolite expression and “BW percent change” and “Treatment” were included as predictors. Proteins/metabolites with a p-value < 0.01 for a given coefficient were considered significantly associated with the coefficient.

**Proteomics Analysis.** Liver tissue was placed in vials containing 1.4 mm zirconia beads and taken up in 500 µL of PreOmics iST buffer (PreOmics). In a cold room set to 4 °C, tissues were homogenized utilizing a bead Ruptor 12 by shaking for 30 seconds for a total of 5 times using ‘high’ setting. Lysate was normalized using BCA assay (Thermo Fisher Scientific), and subsequently incubated at 95 °C for 10 min for reduction, and alkylation of proteins. A total of 50 μg from each sample was subjected to enzymatic cleavage for 3 h by adding equal amounts of endoproteinase Lys-C and trypsin (ThermoFisher Scientific, # A40009) in a 1:50 (wt/wt) enzyme:protein ratio. De-salting and purification were performed according to the PreOmics iST protocol on a styrene divinylbenzene reversed-phase sulfonate sorbent. Purified peptides were vacuum-centrifuged to dryness and reconstituted in double-distilled water with 2 vol% ACN and 0.1 vol% formic acid (FA) for single-run LC-MS analysis.

Peptides were loaded onto a ReproSil-Pur 120 C18AQ 1.9 µm in-house packed to a 5 µm tip 75u ID × 360u × 50 cm column using Thermo EASY-nLC 1200 coupled online to an Exploris 480 Mass Spectrometer equipped with a Nanospray Flex Ion Source integrated with a column oven (PRSO-V1, Sonation) maintained at 50 °C. Peptides were separated using a nonlinear gradient: Mobile phase A was 0.1 vol% FA and 3 vol% acetonitrile (ACN) in water, Mobile phase B was 90 vol% ACN and 0.1 vol % FA. The gradient was operated at 250 nL/min flowing 3 vol% B for 3 min, 3 to 5 vol% B over 3 min, 5 to 17 vol % B over 120 min, 17 to 24 vol % B over 26 min, 24 to 30 vol% B over 14 min, 30 to 85 vol% B over 3 min.

The Orbitrap Exploris 480 spray voltage was set to 2200V, funnel RF at 40%, and heated capillary temperature at 300 °C. Method timeline experiment consisted of 3x MS1 scan and 3x tMS2 scan utilizing thee FAIMS CVs (-45, -55, -75). MS1 scan was operated at 120k resolution, 400 to 1000 m/z scan range, 300% AGC target, and 54 ms IT. tMS2 spanned 400 to 1000 m/z space; a total of 75 MS2 variable windows were used taken into account heavily dense peptide regions. Data was acquired at a resolution of 15k, with normalized collision energy of 28%, 1500% AGC target with 30ms IT. Loop control for each tMS2 scan experiment was set to time (1.5sec) such that each FAIMS CV will acquire for no more than 1.5 seconds to obtain sufficient MS1 points per peak.

Data was analyzed using Spectronaut V15.7.2 (Biognosys AG) direct DIA analysis using a isoform Rat database (UniProt downloaded 22.02.2021) consisting of 9,746 protein entries utilizing the Pulsar search engine. Analysis settings were maintained to factory settings where identification was set to 1% false discovery rate (FDR) for precursor and protein level. FDR was calculated with Spectronaut algorithm based on fraction of negative control/synthetic decoy peptides in the samples. Quantification was conducted on MS1 level for specific digest type of Trypsin/P. Static modifications of carbamidomethyl (+57.021 Da) on cystines and dynamic modification of N-terminal acetylation (+42.011 Da) and oxidation (+15.995 Da) of methionine was set.

**Liver MSI.** Tissue samples were snap frozen in dry ice cooled isopentane. Frozen samples were embedded in a HPMA/PVP hydrogel as previously detailed (Dannhorn et al. 2020). Tissue sectioning was performed using a CM1950 cryostat microtome (Leica Biosystems, Nussloch, Germany) at a section thickness of 10 µm. The tissue sections for desorption electrospray ionisation MSI (DESI-MSI), and matrix assisted laser desorption ionisation (MALDI-MSI) were immediately thaw mounted onto Superfrost or indium tin oxide coated microscope slides respectively. Tissue sections for positive ion mode DESI-MSI were washed with xylene for 1 minute to reduce ion suppression of metabolites of interest.

MSI analysis of most metabolites was performed by DESI-MSI and analysis of glutathione and glutathione disulphide was performed with MALDI-MSI. DESI-MSI analysis was carried out using a Q-Exactive mass spectrometer (Thermo Scientific, Bremen, Germany) equipped with an automated 2D-DESI ion source (Prosolia Inc., Indianapolis, IN, USA). Experiments were carried out in both positive and negative mode with a mass range up to a m/z of 1000, with a nominal mass resolution of 70,000. The injection time was fixed to 150 ms resulting in a scan rate of 3.8 pixel/s. The spatial resolution was adapted between experiments to allow acquisition of the data for all directly compared samples within a single experiment of 48 h, with pixel sizes ranging from 75-100 µm. The sprayer was operated with a mixture of 95% methanol, 5% water delivered with a flow rate of 1.5 µL/min and nebulized with nitrogen at a backpressure of 6 bar. The resulting .raw files were converted into .mzML files using ProteoWizard msConvert (version 3.0.4043)(Adusumilli and Mallick 2017) and subsequently compiled to an .imzML file (imzML converter version 1.3) (Race et al. 2012). All subsequent data processing was performed in SCiLS Lab (version 2021b, Bruker Daltonik, Bremen, Germany).

MALDI-MSI analysis was performed on a RapifleX Tissuetyper (Bruker Daltonik, Bremen, Germany) operated in negative mode. The matrix used was 9-Aminoacridine (9-AA) with a concentration of 10mg/ml prepared in 80:20 methanol:water. The matrix was deposited using an automated spray system at 75°C, with 6 passes, 80 µL/min flow rate, 1200 mm/min velocity and a gas pressure of 8 psi (M3-Sprayer, HTX technologies, Chapel Hill, NC, USA). MALDI experiments were performed with a spatial resolution of 50 µm. A total of 400 laser shots were summed up per pixel to give the final spectra. For all experiments the laser was operated with a frequency of 5 kHz. All raw data was directly uploaded and processed in SCiLS lab (Version 2021b) software packages. All DESI data were normalised to root mean squared and MALDI to the total ion current to compensate for signal variation across the course of the experiments. Putative identities of metabolites were assigned based on comparison of observed and theoretical mass. Statistical analysis was performed using GraphPad Prism v9.5.1, using a Kruskal-Wallis test.

**LC-MS Conditions.** For LC-MS analysis 50 µL of plasma was added to 150 µL cold solvent (ACN/MeOH/H_2_O (40/40/20 v/v/v)), incubated for ten minutes at -20 °C and centrifuged for 10 minutes at (878g) 5 µL of supernatant was added to 45 µL of ACN/MeOH/H_2_O with 2.5 µL of deuterated citrulline as an internal standard. Tissue samples (liver, brain, heart) were extracted with 1 mL of solvent/100 mg tissue (PrecellysCK28K-2ml tube). Tissues were homogenised/extracted in 3x20 sec shake at 5500 rpm and centrifuged for 5 min at 10,621 g. After centrifugation a maximum of 800 µL clear supernatant was transferred to cryovials and the same amount of extraction solvent used in the first step was added to tissue precipitate. Extraction procedure was repeated as above and clear supernatants combined in the same cryovial. 5 µL of supernatant was added to 45 µL 40:40:20 ACN:MeOH:0.5% formic acid (Aq) with 2.5 µL of deuterated citrulline.

For LCMS analysis, standard curves were spiked to give a calibrated range of 1.0 – 1000 nM for SAM and 1.0 – 2000 for methionine. 5 µL of standard spike solution was added to 45 µL of ACN/MeOH/H2O (40/40/20 v/v/v) and 2.5 µL of deuterated citrulline is added as an internal standard. A further calibration curve was prepared with 5 µL blank mouse plasma, 5 µL of standard and 40 µL of 40:40:20 Acetonitrile:Methanol:0.5% formic acid (Aq) with 2.5 µL of deuterated citrulline was used to investigate matrix suppression effects. Blank plasma was also diluted 1:10 with ACN/MeOH/H2O (40/40/20 v/v/v) and was also analysed to establish systemic biomarker levels.

The samples were analysed by injecting 1 µL of the sample onto a Waters Acquity LC system running Hydrophilic Interaction Chromatography (HILIC) methodology.

**Metabolomic sample preparation and analysis.** Tissue samples (liver, brain, heart) were extracted with 1 mL of ACN/MeOH/H2O 40/40/20 v/v/v per 100 mg material in a PrecellysCK28K-2 mL tube. Tissues were homogenised/extracted in 3x20 sec shake at 5500 rpm and centrifuged for 5 min at 10,621g. After centrifugation a maximum of 800 µL clear supernatant was transferred to cryovials and the same amount of extraction solvent used in the first step was added to tissue precipitate. Extraction procedure was repeated as above and clear supernatants combined in the same cryovial and vial labelled as polar metabolite extract. On the remaining tissue pellet 1ml (per100mg tissue material) of Butanol/MeOH 3/1v/v was added and the above extraction sequence applied. Resulted clear supernatant transferred to a clean cryovial and labelled as lipid extract. All samples kept at the lowest available temperature (0 °C) throughout the extraction process and stored at -80 °C until further analysis.

**Polar metabolite analysis.** Two separate analytical methodologies were used to obtain the metabolic fingerprint of positive and negative ionising metabolites. 10 μL of polar extract transferred to HPLC polypropylene vial and dried on a vaccum evaporator (SpeedVac SPD140DDA) and dry residual resuspended in 25 μl or 50 μL HPLC water for positive or negative analysis respectively. Positive acquisition performed on a TSQ Vantage (Thermo) mass spectrometer operated by Xcaliber 3.0.63 following a scheduled MRM method with ion source parameters spray voltage 3.5kV, capillary temperature 350°C, vaporiser temperature 400°C, Aux gas 20, sheath gas 60. Chromatographic separation was achieved with gradient elution using solvent A: H_2_O with 0.05% heptafluorobutyric acid and 0.05% acetic acid, solvent B: MeOH with solvent mixing at flow rate 0.4 mL/min as follows 0 min 0% B, 0.5 min 0% B, 3 min at 13% B, 4 min at 13% B, 9 min at 50% B, 9.1 min at 100% B, 11.6 min 100% B 11.7 min at 0% B resulting an injection cycle of 15.5 min per sample. Negative acquisition was performed on a 6600 triple TOF (ABSCIEX) mass spectrometer operated by Analyst TF 1.8.1 and scanning the mass range of 70-1000 m/z with source parameters Gas1 60 Gas 2: 50, curtain gas 35, (arbitrary unit), Temperature 500°C, ion spray voltage –4.5kV, declustering potential –10V and collision energy –10. Negative chromatographic resolution obtained on an ion pair method previously described (Michopoulos et al. 2014).

**Table S4: Lipid positive SWATH variable window acquisition method**

| Precursor start m/z | Precursor ion stop m/z | Declustering potential (DP) V | DP spread (V) | Collision energy (V) | Collision energy spread (V) |
| --- | --- | --- | --- | --- | --- |
| 199.5 | 278.3 | 80 | 0 | 35 | 20 |
| 277.3 | 426.7 | 80 | 0 | 35 | 20 |
| 425.7 | 524.7 | 80 | 0 | 35 | 20 |
| 523.7 | 570.2 | 80 | 0 | 40 | 20 |
| 569.2 | 625.5 | 80 | 0 | 40 | 20 |
| 624.5 | 695.5 | 80 | 0 | 40 | 20 |
| 694.5 | 724.9 | 80 | 0 | 45 | 20 |
| 723.9 | 741 | 80 | 0 | 45 | 20 |
| 740 | 754.3 | 80 | 0 | 45 | 20 |
| 753.3 | 765.5 | 80 | 0 | 50 | 20 |
| 764.5 | 776 | 80 | 0 | 50 | 20 |
| 775 | 786.5 | 80 | 0 | 50 | 20 |
| 785.5 | 796.3 | 80 | 0 | 50 | 20 |
| 795.3 | 806.8 | 80 | 0 | 55 | 20 |
| 805.8 | 819.4 | 80 | 0 | 55 | 20 |
| 818.4 | 832.7 | 80 | 0 | 55 | 20 |
| 831.7 | 850.9 | 80 | 0 | 60 | 20 |
| 849.9 | 880.3 | 80 | 0 | 60 | 20 |
| 879.3 | 934.9 | 80 | 0 | 60 | 20 |
| 933.9 | 1497 | 80 | 0 | 60 | 20 |

**Lipid analysis.** For lipid analysis the same chromatographic separation method was used on both negative and positive acquisitions (reference). 10 µL of each of polar and lipid extract mixed on an HPLC polypropylene vial and analysed directly on positive while for negative 30 μL of blank extraction solvent added prior to analysis. Positive data obtained from 100 to 1500m/z on an X500B (ABSCIEX) operated at spray voltage 5.5kV, Gas1: 50, Gas2: 50, Curtain gas: 25, CAD gas: 7, temperature 400°C. Fragment information collected using a 20 variable SWATH acquisition between 50 to 1500m/z starting from percussor mass 200m/z.

Negative data obtained from 100 to 1500m/z on an 6600 triple TOF (ABSCIEX) operated at spray voltage -4.5kV, Gas1: 60, Gas2: 50, Curtain gas: 35, temperature 500 °C. Fragment information collected using a 25 variable SWATH acquisition between 100 to 1500m/z.

**Table S5: Lipid negative SWATH variable window acquisition method**

| Precursor start m/z | Precursor ion stop m/z | Declustering potential (DP) V | DP spread (V) | Collision energy (V) | Collision energy spread (V) |
| --- | --- | --- | --- | --- | --- |
| 99.5 | 290.7 | 20 | 0 | 35 | 15 |
| 289.7 | 457.3 | 20 | 0 | 40 | 15 |
| 456.3 | 579.6 | 20 | 0 | 40 | 15 |
| 578.6 | 678.2 | 20 | 0 | 40 | 15 |
| 677.2 | 705.4 | 20 | 0 | 40 | 15 |
| 704,4 | 721,2 | 20 | 0 | 40 | 15 |
| 720,2 | 733,3 | 20 | 0 | 40 | 15 |
| 732,3 | 745,5 | 20 | 0 | 40 | 15 |
| 744,5 | 756,9 | 20 | 0 | 40 | 15 |
| 755,9 | 766,9 | 20 | 0 | 40 | 15 |
| 765,9 | 777,6 | 20 | 0 | 40 | 15 |
| 776,6 | 787,6 | 20 | 0 | 40 | 15 |
| 786,6 | 798,4 | 20 | 0 | 40 | 15 |
| 797,4 | 808,4 | 20 | 0 | 40 | 15 |
| 807,4 | 819,8 | 20 | 0 | 45 | 20 |
| 818,8 | 832 | 20 | 0 | 45 | 20 |
| 831 | 843,4 | 20 | 0 | 45 | 20 |
| 842,4 | 855,6 | 20 | 0 | 50 | 20 |
| 854,6 | 868,4 | 20 | 0 | 50 | 20 |
| 867,4 | 883,5 | 20 | 0 | 50 | 20 |
| 882,5 | 901,3 | 20 | 0 | 50 | 20 |
| 900,3 | 927,8 | 20 | 0 | 55 | 20 |
| 926,8 | 1028,6 | 20 | 0 | 55 | 20 |
| 1027,6 | 1415,4 | 20 | 0 | 55 | 20 |
| 1414,4 | 1499,8 | 20 | 0 | 55 | 20 |

**LC-MS/MS analysis of AZ’9567 in rat plasma.** Calibration and quality control samples were prepared in rat plasma with a target concentration range for quantification of 0.1 – 100 µmol/L. Sample preparation: 8 µl of plasma were diluted in 92 µl of 5% (w/v) bovine serum albumin in phosphate buffer and gently shaken for 10 min before 5 min centrifugation at 2400 g at 4°C. 25 µL were transferred to new tubes and 150 µl of acetonitrile containing an internal standard were added for protein precipitation. After thorough mixing and centrifugation for 5 min at 2400 g and 4°C, 75 µl of supernatant were transferred into new tubes and diluted in 150 µL water/HCOOH (100:0.2, v/v) then thoroughly vortexed and again centrifuged for 5 min at 2400 g and 4°C. 1.5 µl of the supernatant were injected for LC-MS/MS analysis utilizing an Acquity UPLC BEH C18, 2.1x50 nm, 1.7 µ column at 60° C. Mobile phase A: Acetonitrile/HCOOH (100/0.2, v/v) and B: water/HCCOH (100/0.2, v/v). Flow rate: 0.75 mL/min.

**Table S6: LC Gradient**

| **Time (min)** | **% A** | **% B** |
| --- | --- | --- |
| **Initial** | **20** | **80** |
| **0.50** | **20** | **80** |
| **1.20** | **95** | **5** |
| **1.50** | **95** | **5** |
| **1.80** | **20** | **80** |

**Table S7: MS/MS parameters (API 4500)**

| **Compound** | **Mode** | **Parent ion**  **m/z** | **Daughter ion**  **m/z** | **Dwell**  **msec** | **Cone Voltage** |
| --- | --- | --- | --- | --- | --- |
| **AZ’9567** | ESI +ve | 448.3 | 390.0 | 50 | 5500 |
